# Discovery and preclinical characterization of [^18^F]PI-2620, a next-generation tau PET tracer for the assessment of tau pathology in Alzheimer’s disease and other tauopathies

**DOI:** 10.1007/s00259-019-04397-2

**Published:** 2019-07-01

**Authors:** Heiko Kroth, Felix Oden, Jerome Molette, Hanno Schieferstein, Francesca Capotosti, Andre Mueller, Mathias Berndt, Heribert Schmitt-Willich, Vincent Darmency, Emanuele Gabellieri, Cédric Boudou, Tanja Juergens, Yvan Varisco, Efthymia Vokali, David T. Hickman, Gilles Tamagnan, Andrea Pfeifer, Ludger Dinkelborg, Andreas Muhs, Andrew Stephens

**Affiliations:** 1 AC Immune SA, Lausanne, Switzerland; 2 Life Molecular Imaging, GmbH, Berlin, Germany; 3 Piramal Imaging GmbH, Berlin, Germany; 4 Present Address: Merck KGaA, Darmstadt, Germany; 5 Invicro LLC, New Haven, USA; 6 Present Address: XingImaging LLC, Beijing, China

**Keywords:** Tau, PET, Positron-emission tomography, AD, Alzheimer’s disease, Tauopathies, Fluorine-18, PET tracer, PI-2620

## Abstract

**Purpose:**

Tau deposition is a key pathological feature of Alzheimer’s disease (AD) and other neurodegenerative disorders. The spreading of tau neurofibrillary tangles across defined brain regions corresponds to the observed level of cognitive decline in AD. Positron-emission tomography (PET) has proved to be an important tool for the detection of amyloid-beta (Aβ) aggregates in the brain, and is currently being explored for detection of pathological misfolded tau in AD and other non-AD tauopathies. Several PET tracers targeting tau deposits have been discovered and tested in humans. Limitations have been reported, especially regarding their selectivity.

**Methods:**

In our screening campaign we identified pyrrolo[2,3-*b*:4,5-*c*’]dipyridine core structures with high affinity for aggregated tau. Further characterization showed that compounds containing this moiety had significantly reduced monoamine oxidase A (MAO-A) binding compared to pyrido[4,3-*b*]indole derivatives such as AV-1451.

**Results:**

Here we present preclinical data of all ten fluoropyridine regioisomers attached to the pyrrolo[2,3-*b*:4,5-*c*’]dipyridine scaffold, revealing compounds **4** and **7** with superior properties. The lead candidate [^18^F]PI-2620 (compound **7**) displayed high affinity for tau deposits in AD brain homogenate competition assays. Specific binding to pathological misfolded tau was further demonstrated by autoradiography on AD brain sections (Braak I-VI), Pick’s disease and progressive supranuclear palsy (PSP) pathology, whereas no specific tracer binding was detected on brain slices from non-demented donors. In addition to its high affinity binding to tau aggregates, the compound showed excellent selectivity with no off-target binding to Aβ or MAO-A/B. Good brain uptake and fast washout were observed in healthy mice and non-human primates.

**Conclusions:**

Therefore, [^18^F]PI-2620 was selected for clinical validation.

**Electronic supplementary material:**

The online version of this article (10.1007/s00259-019-04397-2) contains supplementary material, which is available to authorized users.

## Background

**Fig. 1:**
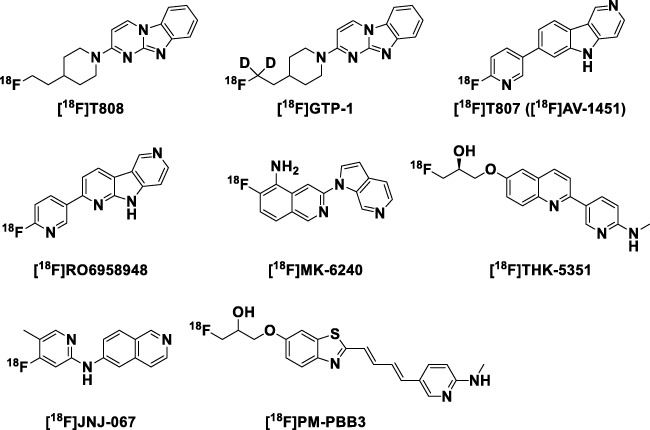
Structures of ^18^F-labeled tau PET tracers

Alzheimer’s disease (AD) is the most common age-related neurodegenerative condition [1, 2]. Although the mechanisms of neurodegeneration in AD are not yet fully understood, the major pathological characteristics are plaques, composed of beta-amyloid (Aβ) peptides, and neurofibrillary tangles (NFTs), composed of hyperphosphorylated tubulin associated unit (tau) proteins. There are several validated positron-emission tomography (PET) tracers available for the detection of Aβ aggregates in vivo that are recognized as important tools to support the diagnosis and clinical management of AD [3–9]. Aβ plaque formation occurs early in the disease process before the onset of clinical symptoms. As such, Aβ plaque load does not correlate well with cognitive performance, while tau tangles have been shown to have a close correlation to neurodegeneration and track better with cognitive decline. Several PET ligands binding tau deposits (Fig. 1) have been developed [3, 10–17]. Detailed reviews of currently studied tracers can already be found in the literature [18–20].

NFTs are comprised of either paired helical filaments (PHFs) or straight filaments (SFs) [21]. Six tau isoforms are expressed in the human brain, which can be classified into two groups, 3-repeat (3R) or 4-repeat (4R), depending on the number of repeats of the microtubule binding domains [22–25].

In AD all six tau isoforms (3R and 4R) are present, and the electron cryo-microscopy (cryo-EM) structure of PHF and SF filament cores reveals that they are made of two identical protofilaments, which adopt a combined cross-β/β-helix structure [23]. The importance of disease-specific folds of tau filaments was recently demonstrated in determining the cryo-EM structure of the neurodegenerative tauopathy Chronic traumatic encephalopathy (CTE) [26]. Though all six tau isoforms are present in AD and CTE, the tau filament folds are different, indicating that the same protein sequences can lead to different aggregates. Other neurodegenerative tauopathies include progressive nuclear palsy (PSP), corticobasal degeneration (CBD) and Pick’s disease (PiD), all of which lack Aβ plaques [23]. In contrast to AD, the aggregated tau proteins of PSP and CBD contain only the 4R tau isoform, whereas the aggregated tau protein in Pick bodies of PiD contain only the 3R tau isoform [27]. The cryo-EM structure of tau filaments from PiD shows a novel fold of 3R tau [28], which is different compared to the 3R/4R-fold found in AD and CTE.

Our goal was to identify a compound which has high affinity to tau aggregates, high selectivity towards pathological tau compared to other targets in the brain, favorable pharmacokinetic properties (fast brain uptake followed by complete washout of any unbound activity), lack of defluorination and lack of potentially image-confounding metabolites. The substitution pattern should support a straightforward introduction of the ^18^F label by routine methods. Binding to both 3R and 4R tau deposits, as well as being able to bind to different tau-folds, would be an advantage [29]. This would allow the detection of tau pathology, not only in AD but also in non-AD tauopathies including PSP and PiD [30, 31]. Due to the mixed pathologies in AD, selectivity over Aβ is required for a tau PET tracer. In addition, ligands should not display significant binding to monoamine oxidase A (MAO-A) and MAO-B, as previous ligands have shown significant binding in vitro which may contribute to off-target binding. Thus, we explicitly built screens against these targets in the discovery pathway.

**Fig. 2:**
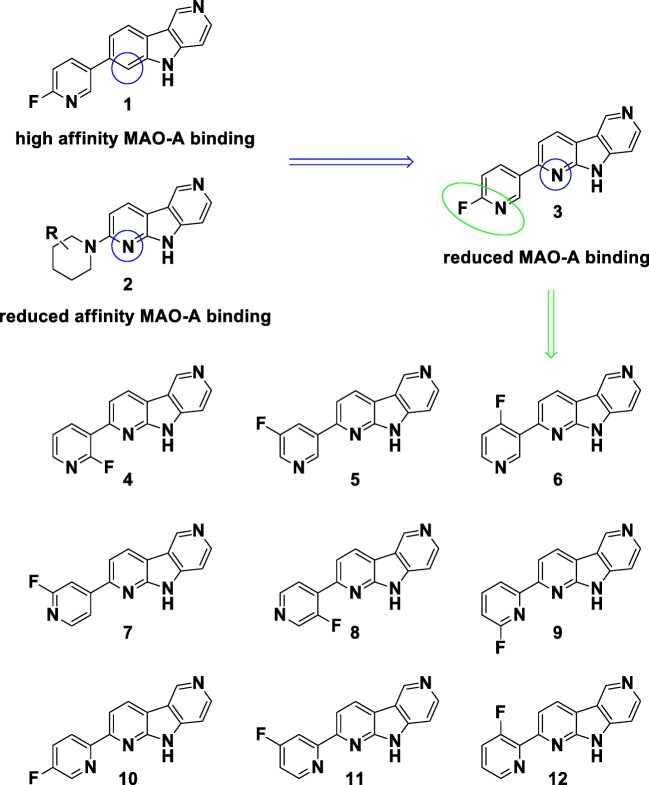
Design of fluoropyridine regioisomers. Data from our screening campaign revealed that both pyrrolo[2,3-*b*:4,5-*c*’]dipyridine and pyrido[4,3-*b*]indole core structures exhibit high affinity for tau, but significantly reduced MAO-A binding was measured for pyrrolo[2,3-*b*:4,5-*c*’]dipyridine core structures only, providing the rationale to investigate all ten fluoropyridine regioisomers in more detail. Compound numbering: **1** (AV-1451), **2**, **3** (RO6958948), **4, 5, 6, 7** (PI-2620), **8, 9, 10, 11** and **12**

We identified pyrrolo[2,3-*b*:4,5-*c*’]dipyridine core structures with high affinity for aggregated tau and significantly reduced MAO-A binding properties compared to pyrido[4,3-*b*]indole derivatives. In order to explore the structure–activity relationship of the fluoropyridine substituent, we synthesized all ten fluoropyridine regioisomers (Fig. 2) to evaluate the impact of the substitution pattern on tau aggregate binding and off-target profile. These ligands were compared to the related compounds **1** (AV-1451) and **3** ([^18^F]RO6958948). All compounds were analyzed for tau aggregate binding and off-target binding to Aβ as well as MAO-A and MAO-B in brain homogenate assays. Selected compounds were subsequently radiolabeled with fluorine-18 and characterized for specific binding by autoradiography (ARG) on fresh-frozen normal, AD and tauopathy brain slices. Brain uptake, washout and bone uptake were assessed by microPET in NMRI mice. In order to overcome the limitation of low resolution of ‘classical’ ARG, selected compounds were radiolabeled with tritium and characterized by micro-autoradiography (micro-ARG). Due to their favorable tau binding, selectivity profile and excellent pharmacokinetic characteristics, compounds **4** and **7** were evaluated in non-human primate (NHP) studies to verify their brain penetration and washout profile. The in vitro and in vivo data, taken together, confirmed compound **[**^**18**^**F]7** as a clinical candidate, which is now called [^18^F]PI-2620.

## Methods

### General chemistry methods

All reagents and solvents were obtained from commercial sources and used without further purification. Proton (^1^H) spectra were recorded on a Bruker DRX-400 MHz NMR spectrometer or on a Bruker AV-400 MHz NMR spectrometer in deuterated solvents. Chemical shifts were reported in δ (ppm) and spin-spin coupling constants as J (Hz) values and signals are designated as follows: (s) singlet, (br-s) broad singlet, (d) doublet, (dd) doublet of doublet, (t) triplet, and (m) multiplet. Mass spectra (MS) were recorded on an Advion CMS mass spectrometer. Flash purification was conducted with a Biotage Isolera One flash purification system using HP-Sil (Biotage) or puriFlash columns (Interchim) and the solvent gradient indicated in the specific examples. Thin-layer chromatography (TLC) was carried out on silica gel plates with UV detection. Preparative thin-layer chromatography (Prep-TLC) was conducted with 0.5 mm or 1 mm silica gel plates (Analtech: Uniplate, F_254_) and the solvents indicated in the specific examples.

### General ^18^F fluorination method

The tracers were synthesized starting from no-carrier-added (n.c.a.) [^18^F]fluoride (1–10 GBq) by ^18^F direct fluorination. The aqueous [^18^F]fluoride solution was trapped on a Sep-Pak Accell Plus QMA light cartridge (Waters) and eluted with a solution K_2_CO_3_/Kryptofix® 2.2.2. The water was removed using a stream of N_2_ at 120 °C and co-evaporated to dryness with acetonitrile (3 × 1 mL). Afterwards, the precursor dissolved in DMSO was added to the dried K[^18^F]F-K_222_ complex. The reaction vial was sealed and heated for 15 min at 120–160 °C (heating block). Hydrochloric acid was then added for deprotection and the mixture was stirred for another 10 min at 110 °C. After neutralization using sodium hydroxide solution and ammonium formate buffer, the mixture was trapped on a C-18 Plus cartridge (Waters). The cartridge was washed with water (5 mL), eluted with acetonitrile and subsequently, the crude product was purified via semi-preparative HPLC (ACE 5 C18; 250 × 10 mm; 10–80% acetonitrile in 0.05 M ammonium formate, 5 mL/min). The isolated tracer was diluted with water (25 mL), trapped on a C-18 Plus cartridge (Waters), washed with water (5 mL), eluted with ethanol (1 mL) and formulated in saline.

[^18^F]fluoroethylharmine, and **[**^**18**^**F]1** (AV-1451) were obtained according to previously described methods [32, 33]. [^3^H]PiB and [^3^H]deprenyl were obtained from Novandi Chemistry AB, Sweden.

### Competition assays

Human or mouse brain homogenates (20 μg/well), recombinant K18 fibrils or PHFs (0.2 μg/well) were incubated with [^**18**^**F**]**3** for screening purposes, as well as **[**^**18**^**F]7** and non-radioactive test compound(s) **1, 3–12** ranging from 0.61 nM to 1000 nM for 60 min at 37 °C in a 96-well plate. For Aβ selectivity assays, competition against [^3^H]PiB was tested. Binding to MAO-A was evaluated using [^18^F]FEH as reversible MAO-A binder. [^3^H]deprenyl competition was used to assess MAO-B binding.

In general, assays were performed in PBS containing 0.1% BSA and 2% DMSO. Non-specific binding (NSB) was determined with samples containing radiolabeled tool compound in the presence of assay buffer without biological substrate and competitor. After incubation, samples were filtered under vacuum on equilibrated GF/B UniFilter plates (PerkinElmer) using the FilterMate 196 (PerkinElmer). Afterwards, filters were washed twice with 200 μL chilled buffer. Top and bottom sides of filter plates were sealed, and an imaging plate was placed on top of the filter plates and exposed for 30 min to O/N. Imaging plates were scanned using the BASReader 5000 (Fuji) and quantified with the AIDA software. Specific binding was calculated by subtracting the NSB signal from measured sample signals. The unblocked radiolabeled tracer signal was defined as total binding (TOTB). pIC_50_ values were calculated using Prism V7 (GraphPad).

Human tissues used in this study were commercially obtained from Tissue Solutions Ltd., Glasgow, UK.

### Autoradiography

Frozen 18 μm thick human brain slices were examined by autoradiography. All slides were equilibrated for at least 1 h in PBS solution prior to use in the experiment. Each brain section was covered with a solution of the ^18^F-labeled tracer in buffer. To determine non-specific binding (NSB) and for blocking experiments, non-radioactive test compounds were used in excess (10 μM) and mixed with the ^18^F-labeled tracer (0.8–2.4 kBq/μL). Brain sections were incubated with tracer solution at room temperature for 1 h in a humidity chamber, drained thereafter and placed in a slide holder. Slides were washed sequentially with PBS for 1 min; 70% EtOH in PBS for 2 min; 30% EtOH in PBS for 1 min; and PBS for 1 min. Slides were allowed to air-dry before being placed under Fuji imaging plates in imaging boxes for 30 min to overnight exposure. Imaging plates were scanned using BASReader 5000 (Fuji) and resulting images were analyzed using AIDA software.

### Micro-autoradiography

Micro-autoradiography (micro-ARG) was performed on frozen human brain sections that were fixed for 15 min at 4 °C with 4% formaldehyde (Sigma, 252,549). Frozen tissue from the entorhinal cortex brain region of an AD donor was purchased from an external provider. For the PSP tissue (superior temporal gyrus region), the brain samples were obtained from the Netherlands Brain Bank (NBB), Netherlands Institute for Neuroscience, Amsterdam (open access.www.brainbank.nl). All material has been collected from donors for or from whom a written informed consent for a brain autopsy and the use of the material and clinical information for research purposes had been obtained by the NBB. Sections were incubated with [^3^H]PI-2620 (90 nM) in buffer, either alone or together with excess of non-radioactive compound **7** (5 μM) for 1 h at room temperature. Sections were then washed as follows: first, in ice-cold buffer for 1 min, then, in ice-cold 70% ethanol twice for 1 min, in ice-cold buffer for 1 min and finally, rinsed shortly in ice-cold distilled water. Sections were subsequently dried for 1 h under a stream of air and then exposed to Ilford Nuclear Emulsion Type K5 (Agar Scientific, AGP9281) in a light-proof slide storage box at 4 °C. After 5 days, the sections were developed by immersing them successively in the following solutions: in Ilford Phenisol Developer (1:5 dilution in water, Agar Scientific, AGP9106), in Ilfostop Stop solution (1:20 dilution in water, Agar Scientific, AGP9104), in Ilford Hypam Fixer (1:5 dilution in H_2_O, Agar Scientific, AGP9183) and rinsed with water according to the manufacturer’s instructions.

When indicated, immunostaining was also performed on the same section. Sections were saturated and permeabilized in blocking buffer (PBS, 10% NGS, 0.25% Triton X-100) for 1 h at room temperature and then incubated overnight at 4 °C with a mouse conformation-dependent anti-tau antibody (MC1, kindly provided by Peter Davies, Northwell, USA). The primary antibody was diluted at 1/250 in PBS, 5% NGS, 0.25% Triton X-100. The following day, sections were washed three times for 5 min with 1X PBS before incubation with a secondary (goat) anti-mouse antibody labeled with AlexaFluor647 (Jackson, 115–605-166) diluted 1/500 in PBS for 45 min at room temperature, and further washed three times in PBS. Sections were mounted using ProLong Gold Antifade reagent (Invitrogen P36930) and imaged with a Pannoramic 250 Slide Scanner (3DHistech; Hungary) in both bright-field and immunofluorescence mode.

### Immunohistochemistry (IHC)

Frozen human brain sections were used for histological analysis. Antibodies used were mouse anti-phospho-tau (clone AT8; Thermo Fisher #MN1020), mouse anti-3R-tau (clone 8E6/C11, Millipore #05–803), mouse anti-4R-tau (hybridoma supernatant of clone ET-3, kindly provided by Peter Davies, Northwell, USA), and biotin-conjugated donkey anti-mouse IgG (Polyclonal, Jackson #715–065-151). Sections were washed with TBS for 5 min, blocked with TBS including 3% donkey serum for 30 min and stained with primary antibodies at 4 °C in a humidified chamber overnight. Thereafter, slides were washed three times in TBS for 5 min, blocked with TBS including 3% donkey serum, and incubated with secondary antibody at RT for 1 h. Upon washing (3 × 5 min in TBS), slides were incubated with peroxidase conjugated streptavidin (Jackson #016–030-084) at RT for 30 min and subsequently washed (3 × 5 min TBS) and stained with AEC Single/Plus (abcam #103742) for 15 min. Staining was stopped by incubation in deionized water for 2 min. Slides were mounted in Aqua-Poly/Mount solution (Polysciences #18606), scanned (VMscope, Germany) and analyzed using CaseViewer 2.1 software.

### PET imaging

PET imaging studies on healthy NMRI mice were performed using the Inveon small animal PET/CT scanner (Siemens, Knoxville, TN). Animals were anesthetized by ventilation anesthesia using an isoflurane oxygen mixture (100 mL/min, 2–2.5% isoflurane). Approximately 100 μL of tracer solution containing an activity of 8–10 MBq in NaCl (10% EtOH) was injected intravenously (i.v.) into animals (*n* = 1–5, female, 20–35 g) via the tail vein. PET scans were initiated at the time of injection of the tracer, and PET data were collected for 60 min. The amount of activity in different regions was quantified using region of interest (ROI) analysis. The analyses were done using Inveon Research Workplace (Siemens, Knoxville, TN). Results were reported as percent injected dose per gram tissue (%ID/g).

One initial brain PET imaging study was conducted with compounds **4** and **7** in the non-human primate (NHP) species rhesus macaque (*Macaca mulatta*) at Invicro LLC (MNI), New Haven, CT, USA. **[**^**18**^**F]4** (184 MBq) or **[**^**18**^**F]7** (181 MBq) was injected i.v. into an anesthetized animal (female, 7.9 kg; 0.01 mg/kg) with intramuscular ketamine (10 mg/kg) and glycopyrrolate administration 2–3 h prior to PET scan. Within the camera, ventilation anesthesia with 1.75% isoflurane was applied. The two studies were performed with the same animal, 3 weeks apart. PET data were acquired for 240 min p.i., and the amount of activity in different brain regions was quantified using ROI analysis.

## Results

### Chemistry

To enable in vitro and in vivo evaluation, all non-radioactive compounds and the corresponding precursors had to be synthesized (details can be found in the supplemental information). The nitro-group was selected as the leaving group for nucleophilic exchange with ^18^F to prepare the corresponding ^18^F-labeled compounds. As heteroarenes containing a nitrogen atom are electron-deficient, the nitro groups at the 2- and 4-position of pyridine are amenable to direct nucleophilic substitution with ^18^F without an additional activating group [34]. In order to improve the solubility of the nitro-precursors, Boc- or trityl protecting group was introduced at pyrrolo[2,3-*b*:4,5-*c*’]dipyridine NH-moiety, as both can be readily cleaved by acid at the end of synthesis.

Thus, the ^18^F-labeling of reference compounds **3**, **4**, and **7** having 2-nitro pyridine moieties worked efficiently with decay-corrected radiochemical yields of 13–20% (radiochemical purity 95–100%). The Boc-protected precursor offered some advantages, such as better solubility compared to the described synthesis of [^**18**^**F]3** using the corresponding unprotected precursor [12]. The ^18^F-labeling of compound **9** with 2-nitro pyridine moiety in the precursor gave lower radiochemical yields of 10% (radiochemical purity 100%). As expected, the ^18^F-labeling of compounds **5**, **6**, **8**, and **12** having a 3-nitro pyridine or 4-nitro pyridine moiety gave poor decay corrected radiochemical yields of ≤3% (radiochemical purity 95–100%). The ^18^F-labeling attempts of compounds **10,** and **11** were not successful. Tritium-labeled compound **7** ([^3^H]PI-2620) was prepared by direct chloro-vs-tritium exchange of precursor **25** with tritium gas in the presence of 10% palladium on charcoal to afford [^3^H]PI-2620 (yield: 444 MBq, purity >99%) with molar activity of 1.72 TBq/mmol.

###  Misfolded tau binding using AD brain homogenate

To assess binding to pathological tau aggregates in AD brain homogenates, **[**^**18**^**F]3** was used as radioligand. AV-1451 (**1**) and the ten fluoropyridine regioisomers were added to measure concentration-dependent displacement of **[**^**18**^**F]3**. Self-competition of compound **3** revealed a pIC_50_ of 8.4. The pIC_50_ for displacement of **[**^**18**^**F]3** by AV-1451 (**1**), was 8.9. Looking at the fluoropyridine regioisomers a significant impact regarding their tau aggregate binding properties was found (Table 1) with 3- and 4-pyridine regioisomers showing higher affinity on tau aggregates compared to the 2-pyridine regioisomers. Compounds **4, 5**, and **7** were comparable to **3** with pIC_50_ > 8. For compound **8** a pIC_50_ value of 8 was measured. For compounds **6**, **9–12** pIC_50_ values between 7.3–7.7 were determined, revealing only a moderate binding affinity to pathological tau aggregates.

**Table 1:** Tau binding, off-target binding radiolabeling and pharmacokinetic properties

Example	[^18^F]3 pIC_50_^a^	[^3^H]PiB ß-amyloid pIC_50_^a^	[^18^F]FEH MAO-A pIC_50_^a^	[^3^H]Deprenyl MAO-B pIC_50_^c^	Radiolabeling^d^	Mouse PK			
						Brain uptake^e^ [% ID/g]	Washout^f^	Washout^g^	Defluorination^h^ [% ID/g]
1 (AV-1451)	8.9	< 6	7.7	6.8	++	5.3	6.5	6.8	4.0
3 (RO6958948)	8.4	< 6	6.3	< 6	++	5.7	10.9	10.3	6.2
4	8.4	< 6	< 6	< 6	++	5.8	22	25	No^*i*^
5	8.2	< 6	< 6	< 6	○	5.3	9.6	10.2	2.9
6	7.3	< 6	< 6	< 6	○	8.2	14.5	14.4	4.8
7 (PI-2620)	8.5	< 6	<6	< 6	++	5.9	16.6	24.3	No^*i*^
8	8.0	< 6	6.1	< 6	○	4.0	19.4	24.1	No^*i*^
9	7.4	6.4	6.6	6.5	+	8.5	7.0	6.8	11.1
10	7.7	< 6	7.1	6.5	−	NA^*j*^	NA^*j*^	NA^*j*^	NA^*j*^
11	7.5	6.6	< 6	< 6	–	NA^*j*^	NA^*j*^	NA^*j*^	NA^*j*^
12	7.4	< 6	< 6	< 6	○	2.1	3.6	2.6	6.2

^a^AD brain homogenate; ^*b*^ Mouse brain homogenate; ^*c*^ NDC brain homogenate; ^*d*^ − no ^18^F-labeling, ○ poor, + moderate, ++ good, NA: precursor prepared as described; ^*e*^ peak uptake (injected dose per gram brain; ID/g); ^*f*^ ratio of peak uptake divided by peak at 30 min; ^*g*^ ratio of peak uptake divided by peak at 60 min, ^*h* 18^F bone uptake in shoulder joint at 60 min; ^*i*^ no defluorination detected; ^*j*^ NA: not available due to failed ^18^F-labeling

### Off-target binding

To test for selectivity of compounds to misfolded tau over Aβ deposits, the tritiated Pittsburgh compound B ([^3^H]PiB) was used in competition assays using AD brain homogenate. Compounds **3**, **4**, **5**, **6**, **7**, **8** and **10** did not compete with [^3^H]PiB in this assay, showing pIC_50_ values below 6. In contrast, compounds **9** and **11** showed measurable PiB competition resulting in pIC_50_ values of 6.4 and 6.6, respectively, whereas PiB self-competition revealed a pIC_50_ of 7.7.

To evaluate off-target binding to MAO-A, a displacement assay with the selective, reversible MAO-A binder 2-[^18^F]fluoroethyl harmine ([^18^F]FEH, MAO-A pIC_50_ of 9.3 [33] was established. Mouse brain homogenate was used for this assay, as it does not contain pathological human tau aggregates. This is especially important for this assay, as FEH also binds to misfolded human tau aggregates. Thus, competition of **[**^18^F]FEH in mouse brain homogenate can be attributed solely to MAO-A off-target binding (Table 1).

Compound **1** (AV-1451) showed high affinity binding to MAO-A (pIC_50_ = 7.7) in mouse brain homogenate, which was similar to the high affinity, displaceable binding observed for [^3^H]AV-1451 in non-AD cortical brain homogenates (K_d_ ~5–10 nM) [35]. Compound **3** showed significantly reduced MAO-A binding compared to AV-1451 (**1**), with a pIC_50_ of 6.3. The MAO-A binding was further reduced to a pIC_50_ of 6.1 for compound **8**. Compounds **4, 5, 6, 7**, **11** and **12** had non-measurable affinity towards MAO-A with pIC_50_ values <6. Compound **10** displayed quite potent MAO-A binding, with a pIC_50_ of 7.12, whereas compound **9** showed moderate MAO-A binding, with an pIC_50_ of 6.6.

To evaluate off-target binding to MAO-B, a displacement assay with the selective, reversible MAO-B binder deprenyl ([^3^H]deprenyl MAO-B pIC_50_ of 7.7 in the same assay) was established. Human brain tissue from a non-demented control (NDC) was used for this assay (Table 1).

Compounds **9** and **10** showed moderate competition of [^3^H]-deprenyl, with a pIC_50_ value of 6.5 for both compounds. All other compounds were not able to significantly compete [^3^H]deprenyl tested at a concentration to 1 μM, resulting in pIC_50_ values <6. The pIC_50_ values for THK5151, a known tau aggregate binder with significant off-target binding to MAO-B [36] and AV-1451 (**1**), were also determined in this assay. The pIC_50_ values were 7.4 for THK5151 and 6.8 for AV-1451 (**1**), respectively.

### In vivo pharmacokinetic profile in mouse and non-human primate (NHP)

The in vivo pharmacokinetic (PK) profiles of **[**^**18**^**F]1** (AV-1451) and **[**^**18**^**F]3** as well as compounds [^**18**^**F]4**, [^**18**^**F]5**, [^**18**^**F]6**, [^**18**^**F]7**, [^**18**^**F]8**, [^**18**^**F]9**, and [^**18**^**F]12** in mice are summarized in Table 1. Due to the unsuccessful radiolabeling of **10** and **11**, the PK profile of [^**18**^**F]10** and [^**18**^**F]11** could not be assessed. The mouse brain PK data in Table 1 showed good brain uptake (>4% ID/g) for **[**^**18**^**F]1** (AV-1451), **[**^**18**^**F]3**, and compounds [^**18**^**F]4-**[^**18**^**F]9**. The uptake was quite fast as the maximum brain concentration was reached within two minutes. In contrast, brain uptake of [^**18**^**F]12** was poor (2.1% ID/g). The initial washout from the mouse brain was assessed by the ratio of peak uptake divided by the peak at 30 min. Compound **[**^**18**^**F]3** and compounds [^**18**^**F]4**, [^**18**^**F]6**, [^**18**^**F]7** and [^**18**^**F]8** showed the fastest initial washout, with a ratio of >10, whereas **[**^**18**^**F]1** (AV-1451) and compounds [^**18**^**F]5**, [^**18**^**F]9** showed a slower initial washout, with a ratio of 6.5 to 9.5. In addition to poor brain uptake, compound [^**18**^**F]12** showed a very poor washout, with a ratio of 2.1 (Table 1). The activity in the brain at 60 min p.i. was measured to evaluate how complete the ^18^F-labeled compound and/or potential ^18^F-labeled metabolites were cleared from the mouse brain. Compounds [^**18**^**F]4**, [^**18**^**F]7** and [^**18**^**F]8** displayed the most complete washout, with a ratio of 24 to 25, indicating a negligible radioactive background signal at 60 min. In contrast, **[**^**18**^**F]1** (AV-1451), **[**^**18**^**F]3** and compounds [^**18**^**F]5**, [^**18**^**F]6**, and [^**18**^**F]9** displayed a higher radioactive background signal after 60 min, with a ratio of 6.8 to 14.4.

While compounds [^**18**^**F]4**, [^**18**^**F]7** and [^**18**^**F]8** showed no bone uptake after 60 min, **[**^**18**^**F]1** (AV-1451) and compounds [^**18**^**F]5**, [^**18**^**F]6** showed a minor to moderate bone uptake of 2.9–4.8% of the injected dose in mice. In contrast, **[**^**18**^**F]3** and compounds [^**18**^**F]9**, [^**18**^**F]12** displayed high to very high bone uptake of 6.2–11% of the injected dose (Table 1). Taken together, regarding their pharmacokinetic profiles in mouse, compounds **4**, **7** and **8** were identified as the best compounds in terms of brain uptake, fast and complete washout from the mouse brain and having no defluorination. Based on the tau binding data obtained with AD brain-derived homogenates and PK data, compounds **4** and **7** were selected for a PK study in NHP rhesus macaque (*Macaca mulatta*) performed at Molecular Neuroimaging LLC (MNI), New Haven, CT, USA. This pilot NHP PET study confirmed the brain penetration and fast washout from the brain observed in mice. Whole brain peak %ID was 2.5% at 3.5. minutes p.i. with a clearance rate of 0.125 min^−1^ for compound **4** and 1.8% at 3.5 min p.i. with a clearance rate of 0.085 min^−1^ for compound **7.**

**Fig. 3:**
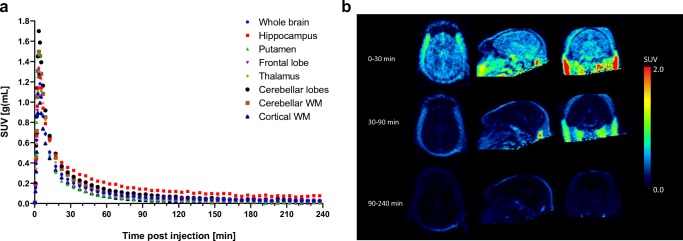
Time activity curves (SUV) for [^18^F]PI-2620 ([^18^F]**7**) in brain regions at baseline in a rhesus macaque (**a**). SUV images at three different time intervals p.i. (**b**)

The fast clearance across all brain regions resulted in standardized uptake values (SUVs) of less than 0.3 at 60 min p.i. for compound **7** (Fig. 3a). The fast washout from the brain is also visualized in Fig. 3b, demonstrating absence of off-target binding for compound **7**.

### Binding characteristics of [^18^F]4 and [^18^F]7

To further evaluate the binding properties of **[**^**18**^**F]4** and **[**^**18**^**F]7** to pathological tau aggregates, additional ligand binding assays were performed. Self-competition experiments revealed pIC_50_ values against isolated PHFs from AD brain sample (Braak IV) of 8.1 and 8.4 (Table 2) for compounds **4** and **7**, respectively. In addition, three different AD brain homogenates (Braak stages V and VI) were tested revealing pIC_50_ values of 8.4 ± 0.1 and 8.5 ± 0.1 for compounds **4** and **7**, respectively (Table 2). The tracers **[**^**18**^**F]4** and **[**^**18**^**F]7** were also evaluated regarding their potential to recognize tau aggregates of non-AD tauopathies. Recombinant human K18 fibrils (depicting 4R pathology) were used in self-competition assays showing a pIC_50_ of 8.4 ± 0.1 for **[**^**18**^**F]7** but no binding of **[**^**18**^**F]4**. However, heparin-induced tau aggregates obtained from recombinant tau protein tend to be polymorphic with different tau protein folds compared to AD, and PiD [36]. In order to verify the lack of binding of **[**^**18**^**F]4** and binding of **[**^**18**^**F]7** to 4R tau aggregates, brain homogenates of two PSP subjects were also used in additional competition assays. Again, no binding of compound **[**^**18**^**F]4** to the substrates could be detected. In contrast, a pIC50 value of 7.7 ± 0.1 was measured for compound **[**^**18**^**F]7**. Both compounds were also tested in a self-competition experiment using a human Pick’s disease brain homogenate. The resulting pIC_50_ values of 8.6 for **[**^**18**^**F]7** and 7.6 for **[**^**18**^**F]4** again favored compound **[**^**18**^**F]7** for further evaluation. Thus, compound **7**, now called [^18^F]PI-2620, demonstrated the potential to bind to both 3R and 4R aggregated tau isoforms as well as to different tau aggregate folds (Table 2).

**Table 2:** Binding characteristics of [^18^F]PI-2620 ([^18^F]7) and [^18^F]4

Biological substrate	[^18^F]PI-2620 (pIC_50_)	[^18^F]4 (pIC_50_)
PHFs	8.4	8.1
AD brains	8.5 ± 0.1	8.4 ± 0.1
K18 fibrils	8.4 ± 0.1	Not detected
PSP brains	7.7 ± 0.1	Not detected
PiD brain	8.6	7.6

### Autoradiography of [^18^F]PI-2620 ([^18^F]7) using AD, NDC and PSP samples

**Fig. 4:**
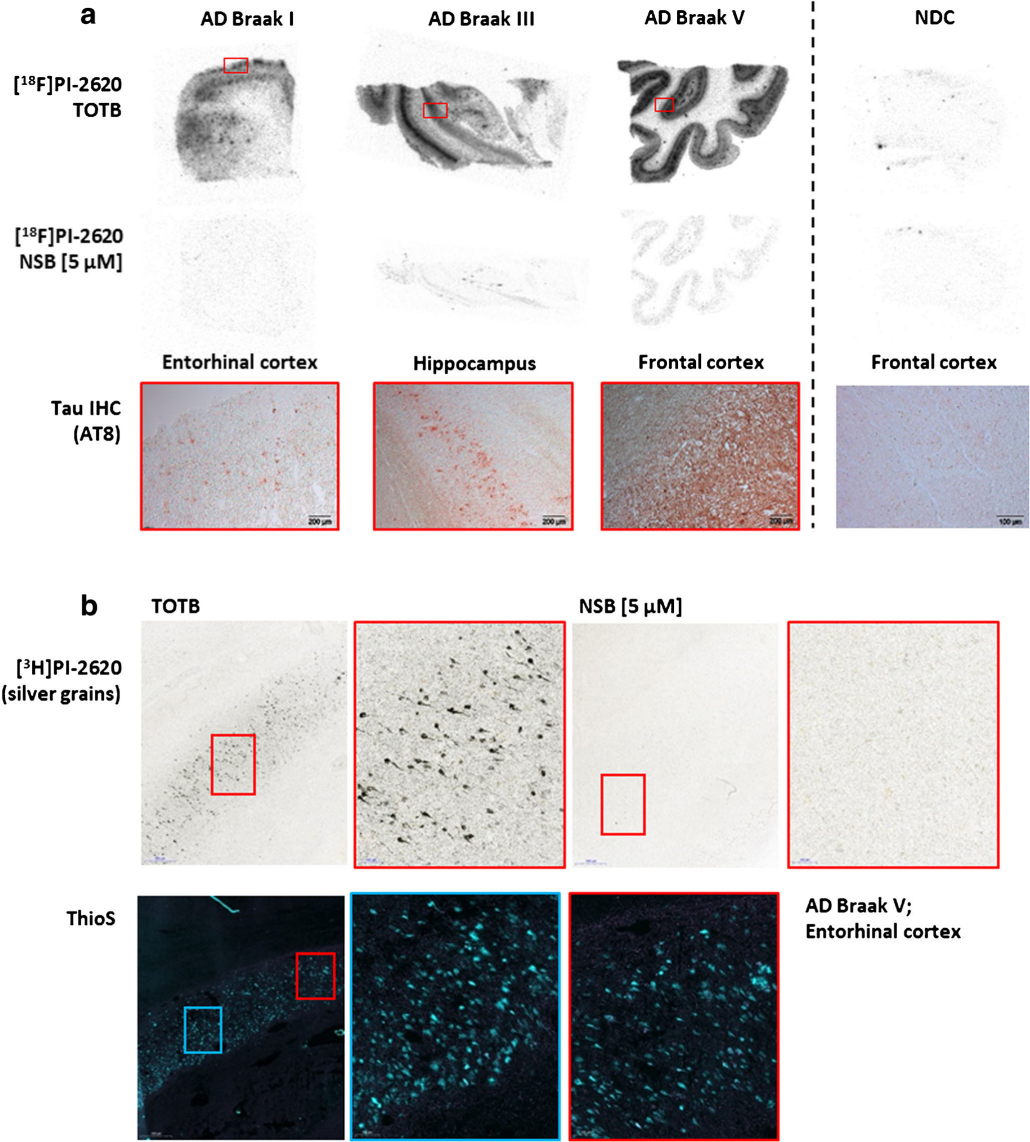
Assessment of specific binding of PI-2620 (**7**) to tau aggregates in human AD brain sections. **a** [^18^F]PI-2620 was tested in ARG on AD patient-derived brain slices staged Braak I, III, and V as well as a non-demented control (NDC). Non-specific binding (NSB) was determined adding 5 μM unlabeled PI-2620. Specific ARG signal correlates with IHC staining on adjacent slices using AT8 antibody. **b** Micro-ARG in entorhinal cortex brain sections from an AD donor with [^3^H]PI-2620 revealed accumulation of silver grains on NFT which is blocked by adding 5 μM unlabeled PI-2620. Thioflavin S staining on adjacent sections correlated with the specific micro-ARG signal. TOTB, total binding; NSB, non-specific binding

**Fig. 5:**
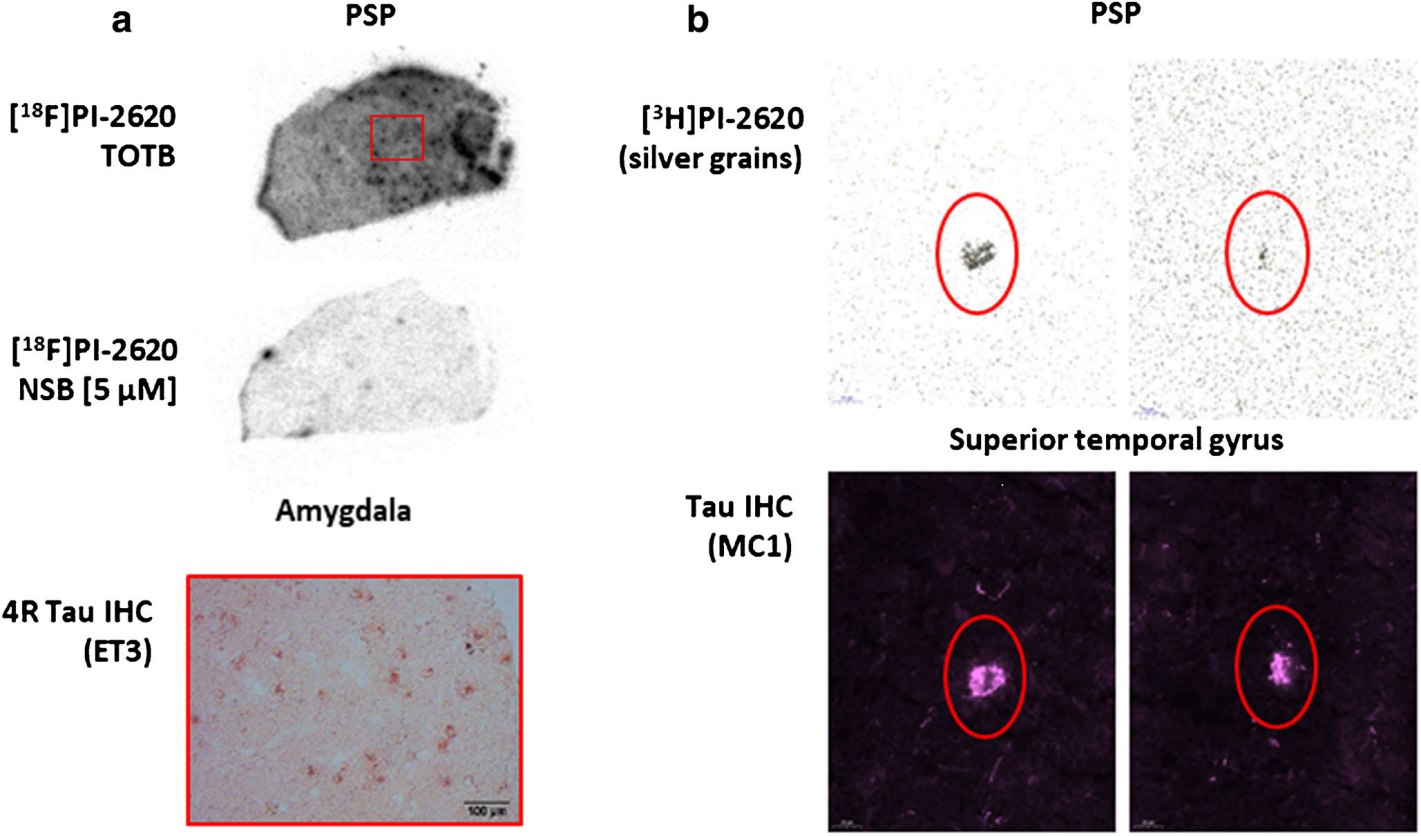
Assessment of specific binding of PI-2620 (**7**) to tau aggregates in human PSP brain sections. **a** ARG of [^18^F]PI-2620 on PSP patient-derived brain slices. Specific ARG signal correlated with IHC staining on adjacent section using the 4R-specific ET3 antibody (kindly provided by Peter Davies, Northwell, USA). **b** Micro-ARG signal on PSP patient-derived brain sections correlated with IHC staining on the same sections using the MC1 antibody

Binding of [^18^F]PI-2620 to AD and non-AD tauopathies was further evaluated by autoradiography using AD brain sections from Braak stages I, III and V and non-demented control (NDC) samples (Fig. 4a). Autoradiography results were compared with immunostaining of identical and/or adjacent brain sections using tau-specific antibodies. In the pathological brain sections analyzed, the area of specific autoradiographic signal of [^18^F]PI-2620 correlated with tau-specific immunostaining. The NDC brain section revealed very low to no off-target binding for [^18^F]PI-2620 in the absence of pathological tau. Additionally, micro-ARG studies were performed with [^3^H]PI-2620 in AD brain sections (Fig. 4b). The micro-ARG signal of accumulating silver grains, generated by [^3^H]PI-2620, revealed a region rich in NFT aggregates in the AD brain section examined, recapitulating the immunofluorescence signal of Thioflavin S staining in an adjacent section. More importantly, the binding of [^3^H]PI-2620 to NFT was specific, since in the presence of compound **7** in excess, the micro-ARG signal was displaced. Using brain tissue slides from a PSP donor, [^18^F]PI-2620 demonstrated selective binding to 4R tau pathology in vitro (Fig. 5a). Furthermore, MC1 immunostaining of a PSP brain section, labeling tau-positive tangle-like structures, colocalized with the micro-ARG signal of [^3^H]PI-2620 in the same section, suggesting target engagement to pathological tau aggregates in PSP tissue (Fig. 5b). However, in the specific brain region examined, very few tau-positive structures of tangle-like size were identified. Due to limitations in resolution, we were not able to detect micro-ARG signals colocalizing with MC-1-positive tau aggregates resembling threads or tufted astrocytes.

## Discussion

We initiated our PET-ligand search by screening the Morphomer™ library to identify small molecules with suitable target to off-target binding properties. Pyrrolo[2,3-*b*:4,5-*c*’]dipyridine and pyrido[4,3-*b*]indole core structures showed high affinity for tau deposits and low off-target binding to Aβ in a human AD brain homogenate assays. Further screening for selectivity using [^18^F]FEH, a reversible MAO-A ligand and [^18^F]deprenyl, a reversible MAO-B ligand, revealed that the 9*H*-pyrrolo[2,3-*b*:4,5-*c*’]dipyridine core can lead to molecules with improved selectivity.

Structure–activity relationship (SAR) analysis around the pyridine substituents has not been previously described. To verify whether the exchange of the 5*H*-pyrido[4,3-*b*]indole core with the 9*H*-pyrrolo[2,3-*b*:4,5-*c*’]dipyridine core would lead to tau PET tracers with high affinity binding to pathological tau aggregates and reduced MAO-A and MAO-B binding, all ten possible fluoropyridine regioisomers were prepared (Fig. 2).

Despite the apparent structural similarities of the regioisomers and comparators **1** (AV-1451) and **3** (RO6958498), the different compounds gave rise to a remarkably diverse set of characteristics. The 2-fluoropyridine isomers **9**, **10**, **11** and **12** displayed lower pIC_50_ values, between 7.3–7.5, compared to 3-pyridine and 4-pyridine regioisomers when evaluated for target affinity to aggregated tau (Table 1). Regioisomers **4**, **5**, and **7** had the highest affinities with pIC_50_ values >8. Compounds **9** and **11** revealed off-target binding to Aβ and were not further pursued. Affinity to MAO-A was found for several compounds (**3, 8**, **9**, and **10)**. The pIC_50_ value for MAO-A binding of compound **1** (AV-1451) was the highest tested (7.7). Measurable off-target binding to MAO-B in a similar assay employing [^3^H]deprenyl was also noted for several compounds (**9**, **10** and AV-1451, **1**). All labeling precursors for the 10 regioisomers were prepared. As compounds **10** and **11** failed several radiolabeling attempts, microPET PK data were assessed only for the remaining 8 regioisomers **3**, **4**, **5**, **6**, **7**, **8**, **9**, **12** and AV-1451 (**1**). While all tested tracers crossed the murine blood brain barrier, the brain uptake values were noticeably different, ranging from 2.1–8.2% ID/g. The two lead compounds, **4** and **7**, had brain uptake values of 5.8% and 5.9% ID/g, respectively. As these two compounds (**4** and **7)** displayed excellent in vitro properties, a pharmacokinetic analysis of compounds **[**^**18**^**F]4** and **[**^**18**^**F]7** in NHP was performed. These NHP data were consistent with the mouse PK and recapitulated the slightly faster washout of compound **4** over **7**. Compound **7** showed slightly higher affinity for misfolded tau derived from AD compared to **4**. Interestingly, compared to **4**, compound **7** displayed superior binding (Table 2) to both 3R and 4R tau aggregates/tau aggregate folds in self-competition experiments employing recombinant K18 tau fibrils as well as human PSP and PiD brain homogenates. Thus, compound **7** was finally chosen for further evaluation in in vitro autoradiography on patient-derived brain sections.

Taken together, the novel PET tracer **[**^**18**^**F]7**, now called [^18^F]PI-2620, exhibited high affinity to pathological tau aggregates within the low nanomolar range shown in ligand binding assays using brain homogenates and recombinant K18 tau fibrils (Table 2). Moreover, PI-2620 was very selective for pathological tau aggregates with no relevant off-target binding towards Aβ, MAO-A, and MAO-B. In autoradiography experiments, selective binding of [^18^F]PI-2620 to pathological tau present in Braak I, III and V human brain sections was observed (Fig. 4). In contrast, ligand binding assays using NDC brain tissues revealed an extremely low background binding in brains devoid of pathological misfolded tau. The low off-target binding of PI-2620 was confirmed in NHP studies in which high brain uptake and fast and complete washout was observed, with no obvious off-target binding (Fig. 3). Brain kinetic data in animal models do not suggest the presence of a brain penetrating lipophilic metabolite, and the analyses of tracer metabolism will be further described in subsequent clinical publications. In in vitro studies [^18^F]PI-2620 demonstrated its potential to bind to pathological tau aggregates/aggregate folds in both AD and non-AD tauopathies using AD and PSP brain sections (Figs. 4 and 5) as well as brain homogenates of PSP and PiD patients (Table 2). Off target binding to neuromelanin- and melanin-containing cells, including pigmented neurons in the substantia nigra, was observed for various tau PET tracers, including AV-1451 (**1**) [37], and MK-6240 [38]. The same was observed for PI-2620 in the pars compacta of human sections from the substantia nigra (Suppl. Fig. 1).

The binding of tau PET ligands to 4R tau aggregates present in PSP and corticobasal degeneration (CBD) is controversial and has been quite challenging. Several tau PET ligands have been reported not to bind to tau deposits in PSP by autoradiography [13, 37, 39]. Schonhaut et al. reported a statistical increase in [18F]AV-1451 (**1**) uptake in some brain regions including globus pallidus in PSP patients in a clinical trial, although the clinical meaningfulness remains unclear [40]. There are several possible reasons: 1) low affinity of the ligands for 4R tau aggregates/aggregate folds, 2) low density of tau aggregates in PSP tissues, 3) difficulty in distinguishing specific from non-specific binding in basal-ganglia structures, and 4) heterogeneity of tau aggregation and/or tau aggregate folds in 4R tauopathies. From the preclinical data presented here, PI-2620 clearly binds to tau aggregates/aggregate folds in PSP. Clinical data are required to establish the utility of PI-2620 in non-AD tauopathies.

Thus, [^18^F]PI-2620 is currently being evaluated in several clinical trials to define its pharmacokinetic properties and its metabolic profile in humans to confirm its suitability for imaging pathological tau aggregates/aggregate folds in AD and non-AD tauopathies in vivo.

## Electronic supplementary material

Suppl. Fig. 1 (DOCX 292 kb)

ESM 1 (DOCX 2275 kb)
