# Supplementary material for: Discovery and preclinical characterization of [18F]PI-2620, a next-generation tau PET tracer for the assessment of tau pathology in Alzheimer’s disease and other tauopathies
Source: Eur J Nucl Med Mol Imaging. 2019 Jul 1;46(10):2178–89. doi: 10.1007/s00259-019-04397-2 (PMC6667408; doi:10.1007/s00259-019-04397-2)
Supplement: Supplementary file 1 — (DOCX 292 kb) [file 259_2019_4397_MOESM1_ESM.docx]

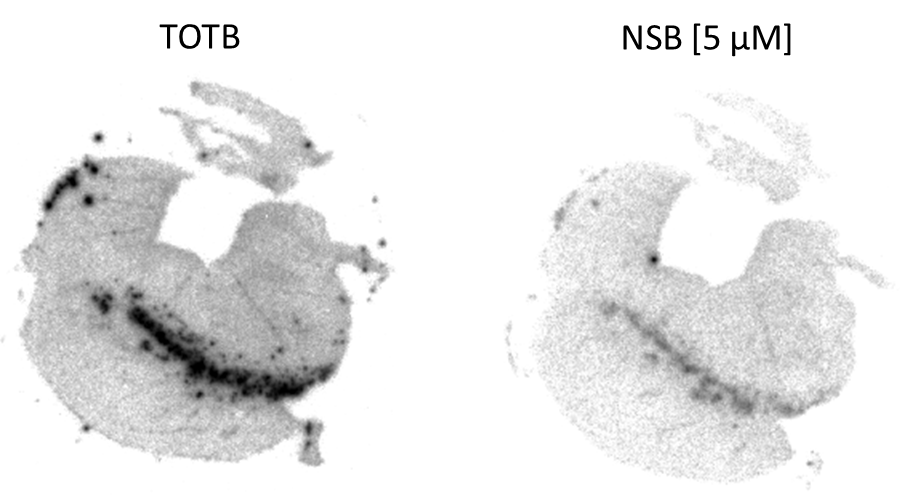


**Supplement Figure 1:** Assessment of specific binding of [^18^F]PI-2620 (**7**) to the pars compacta of human brain sections of the substantia nigra. Non-specific binding (NSB) was determined by addition of 5 µM unlabeled PI-2620.
