# Supplementary material for: Discovery and preclinical characterization of [18F]PI-2620, a next-generation tau PET tracer for the assessment of tau pathology in Alzheimer’s disease and other tauopathies
Source: Eur J Nucl Med Mol Imaging. 2019 Jul 1;46(10):2178–89. doi: 10.1007/s00259-019-04397-2 (PMC6667408; doi:10.1007/s00259-019-04397-2)
Supplement: Supplementary file 2 — (DOCX 2275 kb) [file 259_2019_4397_MOESM2_ESM.docx]

Supplement

**Scheme 1: Synthesis of tricyclic building blocks 16, 17 and 18*^a^***

*^a^* Reagents and conditions: (a) polyphosphoric acid, 160 °C, 20 h. (b) MnO_2_, xylene, 160 °C, 36 h. (c) Trt-Cl, TEA, DMAP, CH_2_Cl_2_, rt, 16 h; (d) Boc_2_O, TEA, DMAP, CH_2_Cl_2_, rt, 16 h..

As the first step to synthesize the different fluoropyridine regioisomers it was necessary to prepare the required tricyclic building blocks **16**, **17** and **18** (Scheme 1) containing a bromo-atom. Commercially available 2-bromo-6-hydrazinylpyridine **13** was reacted with commercially available *tert*-butyl 4-oxopiperidine-1-carboxylate **14** in the presence of polyphosphoric acid under Fischer indole synthesis reaction conditions to afford the tetrahydro-tricycle **15** in 26% yield. Oxidation of **15** under mild conditions with manganese dioxide yielded the fully aromatic tricyclic core **16** with 51% yield, which was converted to the trityl protected derivative **17** in 54% yield. The Boc-protected derivative **18** was obtained in 92% yield.

**Scheme 2: Synthesis of fluoropyridine derivatives 3-12*^a^***

*^a^* Reagents and conditions: (a) Pd(dppf)Cl_2_ x CH_2_Cl_2_, Cs_2_CO_3_, 1,4-dioxane, H_2_O, 3-fluoro-5-(4,4,5,5-tetramethyl-1,3,2-dioxaborolan-2-yl)pyridine, (2-fluoropyridin-4-yl)boronic acid, 2-fluoro-6-(4,4,5,5-tetramethyl-1,3,2-dioxaborolan-2-yl)pyridine, 115-120 °C, 6 h. (b) Pd[P(C_6_H_5_)_3_]_4_, CsF, 1,2-dimethoxyethane, CH_3_OH, (3-fluoropyridin-4-yl)boronic acid 150 °C, 30 min. (c) Pd(dppf)Cl_2_ x CH_2_Cl_2_, 2 M Na_2_CO_3_, *N*,*N*'-dimethylacetamide, (2-fluoropyridin-3-yl)boronic acid, 110 °C, 3 h. (d) Pd(dppf)Cl_2_ x CH_2_Cl_2_, Cs_2_CO_3_, 1,4-dioxane, H_2_O, (6-fluoropyridin-3-yl)boronic acid, 100 °C, 4 h; (e) Pd(dppf)Cl_2_ x CH_2_Cl_2_, 2 M Na_2_CO_3_, *N*,*N*'-dimethylacetamide, 4-fluoro-3-(4,4,5,5-tetramethyl-1,3,2-dioxaborolan-2-yl)pyridine, 110 °C, 22 h. (f) (1) TFA, CH_2_Cl_2_; (2) NaOH. (g) Pd(dppf)Cl_2_ x CH_2_Cl_2_, KOAc, 1,4-dioxane, 4,4,4',4',5,5,5',5'-octamethyl-2,2'-bi(1,3,2-dioxaborolane), 95 °C, 18 h; (h) Pd(dppf)Cl_2_ x CH_2_Cl_2_, Cs_2_CO_3_, 1,4-dioxane, H_2_O, 2-bromo-5-fluoropyridine / 2-bromo-4-fluoropyridine / 2-bromo-3-fluoropyridine, 115 °C, 6 h.

The synthesis of compounds **4**, **5**, **7**, **8** and **9** was accomplished by Suzuki-Miyaura coupling of **16** with the corresponding fluoro-pyridine boronic acid or ester derivatives (Scheme 2). Compounds **5**, **7**, **9** were obtained in moderate to good yields (43-63%) using [1,1′-bis(diphenylphosphino)-ferrocene]dichloropalladium(II) as catalyst, cesium carbonate as base and dioxane/water as solvents. Modified conditions were employed to synthesize **4** by using *N*,*N*’-dimethylacetamide as solvent and sodium carbonate as base (54% yield). For compound **8** cesium fluoride as base and tetrakis(triphenyl-phosphine)palladium(0) as catalyst and 1,2-dimethoxyethane-methanol as solvent mixture were employed using microwave conditions at elevated temperature to afford **8** in 24% yield. The synthesis of compound **3** was already reported using the chloro derivative of **18** to obtain **3** after 2 steps (Suzuki-Miyaura-coupling followed by acid cleavage of the Boc-protecting group) in low overall yield (8.8%)(12). Suzuki-Miyaura coupling of the more reactive bromo-derivative **18** and the corresponding fluoro-pyridine boronic acid allowed the synthesis of reference compound **3** with improved yield (31%) in one step as the Boc-protecting group proved to be labile under these conditions.

All attempts to prepare **6** from **16** and the corresponding fluoro-pyridine boronic ester resulted in the formation of inseparable mixtures of **6**, **16** and byproducts. Employing the trityl protected tricycle **17**, and the optimized reaction conditions for the preparation of **4**, allowed the isolation of the trityl-protected analog in 50% yield, Subsequent acidic cleavage of the trityl-moiety yielded **6** in 47% yield.

The synthesis of compounds **10**, **11** and **12** proved to be more challenging. Thus, the synthesis was modified to prepare the boronic ester derivative **19** *in situ*, followed by Suzuki-Miyaura coupling of the intermediate with the corresponding bromo-fluoro-pyridine derivatives (Scheme 2). After cleavage of the Trityl group of the protected coupling products, compounds **10**, **11**, **12** were obtained in 7%, 2% and 6% overall yield, respectively.

In order to prepare the corresponding precursors, the same chemistry as for the fluoro-derivative was employed, i.e. Suzuki-Miyaura coupling of **17** with commercially available nitro-boronic esters, or Suzuki-Miyaura coupling of the boronic ester intermediate of **17** when bromo/chloro-nitro-pyridine derivatives had to be used. The overall yields of the precursors were quite variable (12-75%), depending on the length of the precursor synthesis (one, or three step).

**Scheme 3: Synthesis of tritium labeling precursor 25*^a^***

*^a^* Reagents and conditions: (a) Pd(dppf)Cl_2_ x CH_2_Cl_2_, K_3_PO_4_, 1,4-dioxane, H_2_O, 80 °C, 5 h. (b) LiHMDS, THF, 90 °C, 2 h. (c) Trt-Cl, TEA, DMAP, CH_2_Cl_2_, rt, 16 h. (d) Pd(dppf)Cl_2_ x CH_2_Cl_2_, Cs_2_CO_3_, 1,4-dioxane, H_2_O, 120 °C, 6 h.

The precursor **25** for the synthesis of tritium labeled **7** was prepared via a 4 step synthesis (Scheme 3). Suzuki-Miyaura coupling of (6-bromo-2-fluoropyridin-3-yl)boronic acid **20** and 2-chloro-5-iodopyridin-4-amine **21** afforded the coupling product **22** in 5% yield. Base catalyzed ring closure afforded the tricyclic building block **23** in 65% yield. Trityl protection of compound **23** afforded **24** in 61% yield. Suzuki-Miyaura coupling of **24** with commercially available (3-chloro-2-fluoropyridin-4-yl)boronic acid afforded the tritylated precursor **25** in 36% yield.

**Experimental procedures, analytical characterization and ^1^H-NMR spectra of compounds 3-12, 15-18, and 22-25**

**Synthesis of tricyclic core building blocks 16, 17, 18**

***Step a: 2-bromo-6,7,8,9-tetrahydro-5*H*-pyrrolo[2,3-*b*:4,5-*c*']dipyridine (15)***

Commercially available 2-bromo-6-hydrazinopyridine **13** (10 g, 53.2 mmol) and commercially available 1-Boc-4-piperidone **14** (10.6 g, 53.2 mmol) were added to a 500 mL flask and mixed to become a homogenous blend. Then polyphosphoric acid (80 g, 115% H_3_PO_4_ basis) was added and the mixture was heated at ~160°C in a sand-bath. At ~120°C the Boc-protecting group was cleaved resulting in foaming of the reaction mixture. After complete Boc-cleavage the foam collapsed and the dark reaction mixture was stirred at ~160°C for 20 hours. The reaction was allowed to cool to room temperature and water (400 mL) was added. The reaction mixture was stirred/sonicated until the gummy material was dissolved. The reaction mixture was then placed in an ice-bath and the pH of the solution was adjusted to pH ~12 by adding solid sodium hydroxide pellets (exothermic). The precipitate was collected by filtration and washed with water (400 mL) to remove salts. The precipitate was dissolved in dichloromethane/methanol (9/1; 1500 mL) by sonication and washed with water (2 x 400 mL) to remove remaining salts and insoluble material. The organic phase was dried over Na_2_SO_4_, filtered and the solvents were removed under reduced pressure. The dark residue was treated with dichloromethane (100 mL), sonicated for 5 minutes and the precipitate was collected by filtration. The precipitate was washed with dichloromethane (40 mL) and air-dried to afford **15** as a beige solid (3.5 g, 26 %). ^1^H-NMR (400 MHz, DMSO-d_6_): δ = 11.5 (br-s, 1H), 7.72 (d, *J*=8.0 Hz, 1H), 7.15 (d, *J*=8.0 Hz, 1H), 3.86-3.82 (m, 2H), 3.03 (t, *J*=5.7 Hz, 2H), 2.71-2.65 (m, 2H).

**S1:** ^1^H-NMR spectra (DMSO-d_6_) of 2-bromo-6,7,8,9-tetrahydro-5*H*-pyrrolo[2,3-*b*:4,5-*c*']dipyridine (**15**)

***Step b: 2-bromo-9*H*-pyrrolo[2,3-*b*:4,5-*c*']dipyridine (16)***

Compound **15** (1.75 g, 6.94 mmol) was suspended in xylene (380 mL) and manganese (IV) oxide (6.62 g, 76.9 mmol) was added. The reaction mixture was then heated at ~160°C in a sand-bath for 36 hours. The cooled reaction mixture was evaporated under reduced pressure, the residue suspended in dichloromethane/methanol (1/1; 400 mL) and stirred at room temperature for 30 minutes. The reaction mixture was then filtered through paper filters to remove the manganese (IV) oxide and the filter washed with methanol (50 mL). The combined filtrates were evaporated under reduced pressure and the dark residue purified by chromatography on silica (50 g HP-SIL-cartridge) using a Biotage Isolera system employing an ethyl acetate/heptane gradient (5/95-100/0) to remove unpolar impurities followed by dichloromethane/methanol (90/10 -> 80/20) to afford **16** as dark yellow solid. The total yield from 2 runs was 1.77 g (51 %). ^1^H-NMR (400 MHz, DMSO-d_6_): δ = 12.52 (br-s, 1H), 9.42 (s, 1H), 8.61 (d, *J*=8.1 Hz, 1H), 8.53 (d, *J*=5.7 Hz, 1H), 7.56-7.52 (m, 2H); MS (ESI): m/z = 247.20/249.20 [M+H]^+^.

**S2:** ^1^H-NMR spectra (DMSO-d_6_) of *2*-bromo-9*H*-pyrrolo[2,3-*b*:4,5-*c*']dipyridine (**16**)

***Step c: 2-bromo-9-trityl-9*H*-pyrrolo[2,3-*b*:4,5-*c*']dipyridine (17)***

To a suspension of compound **16** (0.776 g, 3.13 mmol) in dichloromethane (65 mL) was added triethylamine (1.86 mL, 13 mmol) and trityl-chloride (2.63 g, 9.39 mmol). After the addition of 4-(dimethylamino)-pyridine (0.074 g, 0.608 mmol), the reaction mixture was stirred at room temperature for 16 hours. The reaction mixture was diluted with dichloromethane (150 mL) and water (50 mL). The organic phase was separated, dried over Na_2_SO_4_, filtered and the solvents removed in *vacuo*. The residue was purified on HP-Sil SNAP cartridges (50 g) using a Biotage Isolera One purification system employing an ethyl acetate/n-heptane gradient (5/95 -> 100/0 -> 100/0) to afford **17** as pale yellow solid (0.831 g, 54 %). Unreacted starting material was recovered by flushing the cartridge with ethyl acetate/methanol (90/10) to afford **16** as off-white solid (0.195 g, 25 %). ^1^H-NMR (400 MHz, CDCl_3_) δ = 9.22 (s, 1H), 8.23 (d, *J*=6.2 Hz, 1H), 8.15 (d, *J*=8.1 Hz, 1H), 7.48-7.42 (m, 7H), 7.33-7.22 (m, 12H), 6.41 (dd, *J*=6.2 Hz, *J*=0.9 Hz, 1H); MS (ESI): m/z = 490.03/491.96 [M+H]^+^.

**S3:** ^1^H-NMR spectra (CDCl_3_) of 2-bromo-9-trityl-9*H*-pyrrolo[2,3-*b*:4,5-*c*']dipyridine (**17**)

***Step d: tert-butyl 2-bromo-9H-pyrrolo[2,3-b:4,5-c']dipyridine-9-carboxylate (18)***

To a suspension of compound **16** (0.430 g, 1.73 mmol) in dichloromethane (25 mL) was added triethylamine (1.93 mL, 13.89 mmol) and di-*tert*-butyl dicarbonate (2.27 g, 10.02 mmol). After the addition of 4-(dimethylamino)-pyridine (0.042 g, 0.34 mmol), the reaction mixture was stirred at room temperature for 3 days. The solvents were removed under reduced pressure and the residue was purified on HP-Sil SNAP cartridges (25 g) using a Biotage Isolera One purification system employing an ethyl acetate/n-heptane gradient (5/95 -> 100/0 -> 100/0) to afford **18** as off-white solid (0.558 g, 92 %). ^1^H-NMR (400 MHz, CDCl_3_) δ = 9.28 (s, 1H), 8.73 (d, *J*=5.8 Hz, 1H), 8.23-8.20 (m, 2H), 7.59 (d, , *J*=8.1 Hz, 1H), 1.80 (s, 9H).

**S4:** ^1^H-NMR spectra (CDCl_3_) of tert-butyl 2-bromo-9*H*-pyrrolo[2,3-*b*:4,5-*c*']dipyridine-9-carboxylate (**18**)

**Synthesis of compounds 5, 7, 9**

***Step a: 2-(5-fluoropyridin-3-yl)-9*H*-pyrrolo[2,3-*b*:4,5-*c*']dipyridine (5)***

To a mixture of degassed 1,4-dioxane (4.3 mL) and water (1 mL) in a microwave vial was added [1,1′-bis(diphenylphosphino)ferrocene]dichloropalladium(II), complex with dichloromethane (0.0084 g, 0.01 mmol), followed by compound **16** (0.05 g, 0.2 mmol), 3-fluoro-5-(4,4,5,5-tetramethyl-1,3,2-dioxaborolan-2-yl)pyridine (0.055 g, 0.246 mmol) and cesium carbonate (0.133 g, 0.41 mmol). The reaction mixture was then heated at ~115°C in a sand-bath for 6 hours. The reaction mixture was diluted with ethyl acetate (60 mL) and water (20 mL), the organic phase separated, dried over Na_2_SO_4_, filtered and the solvents evaporated *in vacuo*. The dark residue was purified by chromatography on silica (25 g HP-SIL) using a Biotage Isolera system employing a dichloromethane/methanol gradient (100/0 -> 95/5 -> 90/10 -> 80/20) to afford **5** as off-white solid (0.022 g, 43 %). ^1^H-NMR (400 MHz, DMSO-*d*_6_) δ = 12.45 (br-s, 1H), 9.45 (s, 1H), 9.31 (s, 1H), 8.80 (d, *J*=8.1 Hz, 1H), 8.67 (d, *J*=2.9 Hz, 1H). 8.53 (d, *J*=5.5 Hz, 1H), 8.46-8.40 (m, 1H), 8.11 (d, *J*=8.1 Hz, 1H), 7.52 (d, *J*=5.7 Hz, 1H); MS (ESI): m/z = 265.06 [M+H]^+^.

**S5:** ^1^H-NMR spectra (DMSO-d_6_) of 2-(5-fluoropyridin-3-yl)-9*H*-pyrrolo[2,3-*b*:4,5-*c*']dipyridine (**5**)

***Step a: 2-(2-fluoropyridin-4-yl)-9*H*-pyrrolo[2,3-*b*:4,5-*c*']dipyridine (7)***

To a mixture of degassed 1,4-dioxane (4.3 mL) and water (1 mL) in a microwave vial was added [1,1′-bis(diphenylphosphino)ferrocene]dichloropalladium(II), complex with dichloromethane (0.0084 g, 0.01 mmol), followed by compound **16** (0.05 g, 0.2 mmol), (2-fluoropyridin-4-yl)boronic acid (0.035 g, 0.245 mmol) and cesium carbonate (0.133 g, 0.41 mmol). The reaction mixture was then heated at ~115°C in a sand-bath for 6 hours. The reaction mixture was diluted with ethyl acetate (60 mL) and water (20 mL), the organic phase was separated, dried over Na_2_SO_4_, filtered and the solvents were evaporated *in vacuo*. The dark residue was purified by chromatography on silica (25 g HP-SIL) using a Biotage Isolera system employing a dichloromethane/methanol gradient (100/0 -> 95/5 -> 90/10 -> 80/20) to afford **7** as off-white solid (0.033 g, 63 %). ^1^H-NMR (400 MHz, DMSO-*d*_6_) δ = 12.50 (br-s, 1H), 9.45 (s, 1H), 8.83 (d, *J*=8.1 Hz, 1H), 8.54 (d, *J*=5.7 Hz, 1H), 8.41 (d, *J*=5.3 Hz, 1H), 8.19-8.14 (m, 2H), 7.92 (s, 1H), 7.52 (dd, *J*=5.6 Hz, *J*=1.1 Hz, 1H); MS (ESI): m/z = 265.04 [M+H]^+^.

**S6:** ^1^H-NMR spectra (DMSO-d_6_) of 2-(2-fluoropyridin-4-yl)-9*H*-pyrrolo[2,3-*b*:4,5-*c*']dipyridine (**7**)

***Step a: 2-(6-fluoropyridin-2-yl)-9*H*-pyrrolo[2,3-*b*:4,5-*c*']dipyridine (9)***

To a mixture of degassed 1,4-dioxane (4.3 mL) and water (1 mL) in a microwave vial was added [1,1′-bis(diphenylphosphino)ferrocene]dichloropalladium(II), complex with dichloromethane (0.0084 g, 0.01 mmol), followed by compound **16** (0.05 g, 0.2 mmol), 2-fluoro-6-(4,4,5,5-tetramethyl-1,3,2-dioxaborolan-2-yl)pyridine (0.055 g, 0.246 mmol) and cesium carbonate (0.133 g, 0.41 mmol). The reaction mixture was then heated at ~115°C in a sand-bath for 6 hours. The reaction mixture was diluted with ethyl acetate (60 mL) and water (20 mL), the organic phase separated, dried over Na_2_SO_4_, filtered and the solvents evaporated *in vacuo*. The dark residue was purified by chromatography on silica (25 g HP-SIL) using a Biotage Isolera system employing a dichloromethane/methanol gradient (100/0 -> 95/5 -> 90/10 -> 80/20) to afford **9** as off-white solid (0.033 g, 63 %). ^1^H-NMR (400 MHz, DMSO-*d*_6_) δ = 12.42 (s, 1H), 9.41 (s, 1H), 8.77 (d, *J*=8.1 Hz, 1H), 8.52 (d, *J*=5.6 Hz, 1H), 8.40 (dd, *J*=7.5 Hz, *J*=2.5 Hz, 1H), 8.27 (d, *J*=8.1 Hz, 1H), 8.18 (AB-system, *J*=8.1 Hz, 1H), 7.51 (d, *J*=5.7 Hz, 1H), 7.26 (dd, *J*=8.1 Hz, *J*=2.7 Hz 1H); MS (ESI): m/z = 265.09 [M+H]^+^.

**S7:** ^1^H-NMR spectra (DMSO-d_6_) of 2-(6-fluoropyridin-2-yl)-9*H*-pyrrolo[2,3-*b*:4,5-*c*']dipyridine (**9**)

**Synthesis of compound 8**

***Step b: 2-(3-fluoropyridin-4-yl)-9*H*-pyrrolo[2,3-*b*:4,5-*c*']dipyridine (8)***

In a 5 mL microwave tube were dissolved compound **16** (0.05 g, 0.202 mmol) and (3-fluoropyridin-4-yl)boronic acid (0.0398 g, 0.282 mmol) in 1,2-dimethoxyethane (1.344 mL) and methanol (0.672 mL). Cesium fluoride (0.0306 g, 0.202 mmol) was added and the resulting suspension was degassed for 5 minutes with argon. Then, tetrakis(triphenylphosphine)palladium(0) (0.0419 g, 0.036 mmol) was added, the tube was sealed and the reaction mixture was heated at 150°C in Biotage Initiator microwave for 30 minutes (p~12 bar). The reaction mixture was diluted with ethyl acetate and washed with water and brine. The organic layer was dried over sodium sulfate, filtered and concentrated. The residue was purified by chromatography on silica (10 g HP-SIL) using a Biotage Isolera system employing a dichloromethane/methanol gradient (100/0 -> 80/20) to afford **8** as light brown solid (0.013 g, 24 %). ^1^H-NMR (400 MHz, DMSO-d_6_) δ = 9.46 (s, 1H), 8.82 (d, *J*=8.0 Hz, 1H), 8.77 (d, *J*=3.1 Hz, 1H), 8.63 (d, *J*=5.0 Hz, 1H), 8.56 (d, *J*=5.7 Hz, 1H), 8.09 (dd, *J*=6.9 Hz, *J*=5.0 Hz, 1H), 7.91 (dd, *J*=8.1 Hz, *J*=1.7 Hz, 1H), 7.54 (d, *J*=5.7 Hz, 1H); MS (ESI); m/z = 265.16 [M+H]^+^.

**S8:** ^1^H-NMR spectra (DMSO-d_6_) of 2-(3-fluoropyridin-4-yl)-9*H*-pyrrolo[2,3-*b*:4,5-*c*']dipyridine (**8**)

**Synthesis of compound 4**

***Step c: 2-(2-fluoropyridin-3-yl)-9*H*-pyrrolo[2,3-*b*:4,5-*c*']dipyridine (4)***

In a 5 ml microwave tube compound **16** (0.05 g, 0.202 mmol) and (2-fluoropyridin-3-yl)boronic acid (0.0568 g, 0.403 mmol) were dissolved in *N*,*N*’-dimethylacetamide (2.015 mL). A 2 M aqueous sodium carbonate solution (0.403 mL, 0.806 mmol) was added and the resulting stirring solution was degassed for 5 minutes. [1,1′-bis(diphenylphosphino)-ferrocene]dichloropalladium(II) complex with dichloromethane was added (0.017 g, 0.02 mmol) and the reaction mixture was heated at 110°C for 3 hours. TLC monitoring showed completion of the reaction. The reaction mixture was diluted with ethyl acetate, insoluble material was filtered off, and the filtrate was washed with water and brine. The organic layer was dried with MgSO_4_, filtered and concentrated. The residue was purified by chromatography on silica (10 g HP-SIL) using a Biotage Isolera system employing a dichloromethane/methanol gradient (100/0 -> 90/10) to afford **4** as beige solid (0.028 g, 54 %). %). ^1^H-NMR (400 MHz, DMSO-*d*_6_) δ = 12.45 (br-s, 1H), 9.44 (s, 1H), 8.78 (d, *J*=8.1 Hz, 1H), 8.62-8.57 (m, 1H), 8.54 (d, *J*=5.6 Hz, 1H), 8.37-8.35 (m, 1H), 7.84 (dd, *J*=8.3 Hz, *J*=2.0 Hz, 1H), 7.61-7.57 (m, 1H), 7.57 (d, *J*=5.7 Hz, 1H); MS (ESI): m/z = 265.01 [M+H]^+^.

**S9:** ^1^H-NMR spectra (DMSO-d_6_) of 2-(2-fluoropyridin-3-yl)-9*H*-pyrrolo[2,3-*b*:4,5-*c*']dipyridine (**4**)

**Synthesis of compound 3**

***Step d: 2-(6-fluoropyridin-3-yl)-9*H*-pyrrolo[2,3-*b*:4,5-*c*']dipyridine (3)***

To a mixture of degassed 1,4-dioxane (3 mL) and water (0.7 mL) in a microwave vial was added [1,1′-bis(diphenylphosphino)ferrocene]dichloropalladium(II), complex with dichloro-methane (0.0058 g, 0.007 mmol), followed by **18** (0.05 g, 0.143 mmol), (6-fluoropyridin-3-yl)boronic acid (0.024 g, 0.17 mmol) and cesium carbonate (0.092 g, 0.286 mmol). The reaction mixture was then heated at ~100°C in a sand-bath for 4 hours. The reaction mixture was diluted with ethyl acetate (80 mL) and water (35 mL), the organic phase separated, dried over Na_2_SO_4_, filtered and the solvents evaporated *in vacuo*. The dark residue was purified by chromatography on silica (12 g, puriFlash, Interchim) using a Biotage Isolera system employing a dichloromethane/methanol gradient (100/0 -> 98/2 -> 95/5 -> 90/10 -> 80/20) to afford compound **3** as off-white solid (0.0116 g, 31 %). ^1^H-NMR (400 MHz, DMSO-*d*_6_) δ = 12.40 (br-s, 1H), 9.40 (s, 1H), 9.05 (s, 1H), 8.78-8.70 (m, 2H), 8.51 (d, *J*=5.7 Hz, 1H), 8.02 (d, *J*=8.1 Hz, 1H), 7.50 (d, *J*=7.7 Hz, 1H), 7.36 (dd, *J*=8.6 Hz, *J*=2.9 Hz, 1H); MS (ESI): m/z = 265.09 [M+H]^+^.

**S10:** ^1^H-NMR spectra (DMSO-d_6_) of 2-(6-fluoropyridin-3-yl)-9*H*-pyrrolo[2,3-*b*:4,5-*c*']dipyridine (**3**)

**Synthesis of compound 6**

***2-(4-fluoropyridin-3-yl)-9*H*-pyrrolo[2,3-*b*:4,5-*c*']dipyridine (6)***

***Step e: 2-(4-fluoropyridin-3-yl)-9-trityl-9H-pyrrolo[2,3-b:4,5-c’]dipyridine***

In a 20 ml microwave tube were dissolved compound **17** (0.2 g, 0.408 mmol) and 4-fluoro-3-(4,4,5,5-tetramethyl-1,3,2-dioxaborolan-2-yl)pyridine (0.182 g, 0.816 mmol) in *N*,*N*’-dimethylacetamide (5.10 mL). Sodium carbonate (0.816 ml, 1.631 mmol) was added and the resulting stirring solution was degassed for 5 minutes. Then [1,1′-bis(diphenylphosphino)ferrocene]dichloropalladium(II) complex with dichloromethane was added and the reaction mixture was heated to 110°C for 22 hours. TLC monitoring showed completion of the reaction. The reaction mixture was diluted with dichloromethane, insolubles were filtered out through Celite, and the filtrate was washed with water three times to remove residual amounts of *N*,*N*’-dimethylacetamide. The organic layer was dried MgSO_4_, filtered and concentrated. The residue was purified via Biotage Isolera One (100:0 to 90:10 dichloromethane/methanol; 25g HP-Sil column) to give afford **2-(4-fluoropyridin-3-yl)-9-trityl-9*H*-pyrrolo[2,3-*b*:4,5-*c*’]dipyridine** (0.1036 g; 50 %). ^1^H-NMR (400 MHz, DMSO-d_6_) δ = 9.43 (s, 1H), 8.75 (d, 1H), 8.54 (dd, 1H), 8.26 (d, 1H), 8.17 (d, 1H), 7.83 (dd, 1H), 7.61-7.52 (m, 6H), 7.41 (dd, 1H), 7.35-7.20 (m, 9H), 6.46 (d, 1H); MS (ESI): 507.43, 243.29 [M+H]^+^.

***Step f: 2-(4-fluoropyridin-3-yl)-9H-pyrrolo[2,3-b:4,5-c']dipyridine (6)***

In a 25 ml round bottom flask, **2-(4-fluoropyridin-3-yl)-9-trityl-9*H*-pyrrolo[2,3-*b*:4,5-*c*’]dipyridine** (0.1 g, 0.199 mmol) was dissolved in dichloromethane (1 mL). Trifluoroacetic acid (1 mL) was carefully added and the reaction mixture was stirred for 18 hours at room temperature. After cooling at 0°C, the reaction mixture was quenched to pH = 10 with 2 M sodium hydroxide solution. The resulting suspension was filtered. The reaction mixture was washed with water and brine. The organic was dried over MgSO_4_, filtered and concentrated. The residue was purified by chromatography on silica (10 g HP-SIL) using a Biotage Isolera system employing a dichloromethane/methanol gradient (100/0 -> 90/10) to afford **6** (0.026 g; 47 %). ^1^H-NMR (400 MHz, DMSO-d_6_) δ 12.57 (s, 1H), 9.46 (s, 1H), 9.19 (d, *J*=10.7 Hz, 1H), 8.80 (d, *J*=8.1 Hz, 1H), 8.73-8.67 (m, 1H), 8.59-8.52 (m, 1H), 7.81 (d, *J*=8.1 Hz, 1H), 7.60-7.51 (m, 2H); MS (ESI): 265.29 [M+H]^+^.

**S11:** ^1^H-NMR spectra (DMSO-d_6_) of 2-(4-fluoropyridin-3-yl)-9*H*-pyrrolo[2,3-*b*:4,5-c']dipyridine (**6**)

**Synthesis of compounds 10, 11, 12**

***2-(5-fluoropyridin-2-yl)-9*H*-pyrrolo[2,3-*b*:4,5-*c*']dipyridine (10)***

***Steps g, h: 2-(5-fluoropyridin-2-yl)-9-trityl-9H-pyrrolo[2,3-b:4,5-c']dipyridine***

To a mixture of degassed 1,4-dioxane (8 mL) in a microwave vial was added [1,1′-bis(diphenylphosphino)ferrocene]dichloropalladium(II), complex with dichloromethane (0.034 g, 0.04 mmol), followed by compound **17** (0.2 g, 0.4 mmol), bis(pinacolato)diborane (0.112 g, 0.44 mmol) and potassium acetate (0.118 g, 1.2 mmol). The reaction mixture was then heated at ~95°C in a sand-bath for 18 hours. The reaction mixture was diluted with ethyl acetate (100 mL) and water (30 mL), the organic phase separated, dried over Na_2_SO_4_, filtered and the solvents evaporated *in vacuo* to afford the crude boronic ester derivative. The crude boronic ester derivative was dissolved in a mixture of degassed 1,4-dioxane (8.6 mL) and water (2 mL) in a microwave vial. Then [1,1′-bis(diphenylphosphino)ferrocene]dichloro-palladium(II), complex with dichloromethane (0.034 g, 0.04 mmol), 2-bromo-5-fluoropyridine (0.086 g, 0.49 mmol) and cesium carbonate (0.266 g, 0.82 mmol) were added and the reaction mixture was heated at ~115°C in a sand-bath for 6 hours. The reaction mixture was diluted with ethyl acetate (100 mL) and water (30 mL), the organic phase separated, dried over Na_2_SO_4_, filtered and the solvents evaporated *in vacuo*. The dark residue was purified by chromatography on silica (25 g puriFlash, Interchim) using a Biotage Isolera system employing an ethyl acetate/n-heptane gradient (5/95 -> 100/0 -> 100/0) to afford a mixture of ***9-trityl-9H-pyrrolo[2,3-b:4,5-c']dipyridine*** and ***2-(5-fluoropyridin-2-yl)-9-trityl-9H-pyrrolo[2,3-b:4,5-c']dipyridine*** (0.064 g). The mixture was purified by preparative TLC with a loading of ~0.03 g of mixture per 1000 μM Analtech Uniplate (20 x 20 cm) using dichloromethane/acetone (90/10) as mobile phase to afford more polar ***2-(5-fluoropyridin-2-yl)-9-trityl-9H-pyrrolo[2,3-b:4,5-c']dipyridine*** as off-white solid (0.0385 g, 18.5 % for 3 steps). ^1^H-NMR (400 MHz, CDCl_3_) δ = 9.26 (s, 1H), 8.45 (d, *J*=2.7 Hz 1H), 8.38 (AB-system, *J*=8.1 Hz, 2H), 8.25 (d, *J*=6.1 Hz, 1H), 7.62-7.58 (m, 5H), 7.30-7.18 (m, 12H), 6.56 (d, *J*=6.1 Hz, 1H).

***Step f: 2-(5-fluoropyridin-2-yl)-9H-pyrrolo[2,3-b:4,5-c']dipyridine (10)***

Compound ***2-(5-fluoropyridin-2-yl)-9-trityl-9H-pyrrolo[2,3-b:4,5-c']dipyridine*** (0.0385 g, 0.076 mmol) was dissolved in dichloromethane (5 mL) and trifluoroacetic acid (1.2 mL) was added. The reaction mixture was stirred at room temperature for 6 hours. The reaction mixture was diluted with diluted with dichloromethane (50 mL) and water (20 mL). The pH of the aqueous phase was adjusted to pH~12 by the addition of a 1 M aqueous sodium hydroxide solution. The aqueous layer was separated, extracted with dichloromethane (25 mL), the combined organic layer dried over Na_2_SO_4_, filtered and the solvent removed under reduced pressure. The residue was purified by chromatography on silica (10 g HP-SIL-column) using a Biotage Isolera system employing a dichloromethane/methanol gradient (100/0 -> 95/5 -> 90/10) to afford **10** as a white solid (0.0079 g, 39.3 %). ^1^H-NMR (400 MHz, DMSO-d_6_) δ = 12.40 (br-s, 1H), 9.40 (s, 1H), 8.77 (d, *J*=8.1 Hz, 1H), 8.72 (d, *J*=3.0 Hz, 1H), 8.55-8.50 (m, 2H), 8.35 (d, *J*=8.1 Hz, 1H), 7.95-7.90 (m, 1H), 7.51 (d, *J*=5.7 Hz, 1H); MS (ESI): m/z = 265.06 [M+H]^+^.

**S12:** ^1^H-NMR spectra (DMSO-d_6_) of 2-(5-fluoropyridin-2-yl)-9*H*-pyrrolo[2,3-*b*:4,5-*c*']dipyridine (**10**)

***2-(4-fluoropyridin-2-yl)-9*H*-pyrrolo[2,3-*b*:4,5-*c*']dipyridine (11)***

***Steps g, h: 2-(4-fluoropyridin-2-yl)-9-trityl-9H-pyrrolo[2,3-b:4,5-c']dipyridine***

The crude boronic ester (~0.4 mmol) from **step g** was dissolved in a mixture of degassed 1,4-dioxane (8.6 mL) and water (2 mL) in a microwave vial. Then [1,1′-bis(diphenylphosphino)ferrocene]dichloro-palladium(II), complex with dichloromethane (0.034 g, 0.04 mmol), 2-bromo-4-fluoropyridine (0.086 g, 0.49 mmol) and cesium carbonate (0.266 g, 0.82 mmol) were added and the reaction mixture was heated at ~115°C in a sand-bath for 6 hours. The reaction mixture was diluted with ethyl acetate (100 mL) and water (30 mL), the organic phase separated, dried over Na_2_SO_4_, filtered and the solvents evaporated *in vacuo*. The dark residue was purified by chromatography on silica (25 g puriFlash, Interchim) using a Biotage Isolera system employing an ethyl acetate/n-heptane gradient (5/95 -> 100/0 -> 100/0) to afford a mixture of ***9-trityl-9H-pyrrolo[2,3-b:4,5-c']dipyridine*** and ***2-(4-fluoropyridin-2-yl)-9-trityl-9H-pyrrolo[2,3-b:4,5-c']dipyridine*** (0.0489 g). The mixture was dissolved in dichloromethane (5 mL) and trifluoroacetic acid (1.5 mL) was added. The reaction mixture was stirred at room temperature for 6 hours. The reaction mixture was diluted with diluted with dichloromethane (50 mL) and water (20 mL). The pH of the aqueous phase was adjusted to pH~12 by the addition of a 1 M aqueous sodium hydroxide solution. The aqueous layer was separated, extracted with dichloromethane (25 mL), the combined organic layer dried over Na_2_SO_4_, filtered and the solvent removed under reduced pressure. The residue was purified by preparative TLC with a loading of ~0.03 g of mixture per 1000 μM Analtech Uniplate (20 x 20 cm) using dichloromethane/methanol (90/10) as mobile phase to afford ***2-(4-fluoropyridin-2-yl)-9-trityl-9H-pyrrolo[2,3-b:4,5-c']dipyridine*** as off-white solid (0.0145 g, 7 % for 3 steps). ^1^H-NMR (400 MHz, DMSO-d_6_) δ = 9.42 (s, 1H), 8.76 (d, *J*=8.1 Hz, 1H), 8.67 (dd, *J*=8.8 Hz, *J*=5.5 Hz, 1H), 8.35 (d, *J*=8.1 Hz, 1H), 8.27 (d, *J*=6.0 Hz, 1H), 7.67-7.60 (m, 5H), 7.35-7.22 (m, 11H), 6.81 (dd, *J*=11.0 Hz, *J*=2.7 Hz, 1H), 6.60 (d, *J*=6.0 Hz, 1H).

***Step f: 2-(4-fluoropyridin-2-yl)-9H-pyrrolo[2,3-b:4,5-c']dipyridine (11)***

Compound ***2-(4-fluoropyridin-2-yl)-9-trityl-9H-pyrrolo[2,3-b:4,5-c']dipyridine*** (0.0145 g, 0.027 mmol) was dissolved in dichloromethane (3 mL) and trifluoroacetic acid (2 mL) was added. The reaction mixture was stirred at room temperature for 18 hours. The reaction mixture was diluted with diluted with dichloromethane (50 mL) and water (20 mL). The pH of the aqueous phase was adjusted to pH~12 by the addition of a 1 M aqueous sodium hydroxide solution. The aqueous layer was separated, extracted with dichloromethane (25 mL), the combined organic layer dried over Na_2_SO_4_, filtered and the solvent removed under reduced pressure. The residue was purified by chromatography on silica (10 g HP-SIL) using a Biotage Isolera system employing a dichloromethane/methanol gradient (100/0 -> 95/5 -> 90/10) to afford **11** as off-white solid (0.0025 g, 33 %). ^1^H-NMR (400 MHz, DMSO-d_6_) δ = 12.43 (br-s, 1H), 9.45 (s, 1H), 8.82-8.77 (m, 2H), 8.54 (d, *J*=5.7 Hz, 1H), 8.44 (d, *J*=8.1 Hz, 1H), 8.22 (dd, *J*=10.7 Hz, *J*=2.6 Hz, 1H), 7.53 (d, *J*=5.8 Hz 1H), 7.46-7.42 (m, 1H); MS (ESI): m/z = 264.63 [M+H]^+^.

**S13:** ^1^H-NMR spectra (DMSO-d_6_) of 2-(4-fluoropyridin-2-yl)-9*H*-pyrrolo[2,3-*b*:4,5-*c*']dipyridine (**11**)

***2-(3-fluoropyridin-2-yl)-9*H*-pyrrolo[2,3-*b*:4,5-*c*']dipyridine (12)***

***Steps g, h: Mixture of 9-trityl-9H-pyrrolo[2,3-b:4,5-c']dipyridine & 2-(3-fluoropyridin-2-yl)-9-trityl-9H-pyrrolo[2,3-b:4,5-c']dipyridine***

The crude boronic ester (~0.4 mmol) from **step g** was dissolved in a mixture of degassed 1,4-dioxane (8.6 mL) and water (2 mL) in a microwave vial. Then [1,1′-bis(diphenylphosphino)-ferrocene]dichloropalladium(II), complex with dichloromethane (0.034 g, 0.04 mmol), 2-bromo-3-fluoropyridine (0.086 g, 0.49 mmol) and cesium carbonate (0.266 g, 0.82 mmol) were added and the reaction mixture was heated at ~115°C in a sand-bath for 6 hours. The reaction mixture was diluted with ethyl acetate (100 mL) and water (30 mL), the organic phase separated, dried over Na_2_SO_4_, filtered and the solvents evaporated *in vacuo*. The dark residue was purified by chromatography on silica (25 g puriFlash, Interchim) using a Biotage Isolera system employing an ethyl acetate/n-heptane gradient (5/95 -> 100/0 -> 100/0) to afford a mixture of ***9-trityl-9H-pyrrolo[2,3-b:4,5-c']dipyridine*** and ***2-(3-fluoropyridin-2-yl)-9-trityl-9H-pyrrolo[2,3-b:4,5-c']dipyridine*** (0.0586 g).

***Step f: 2-(3-fluoropyridin-2-yl)-9H-pyrrolo[2,3-b:4,5-c']dipyridine (12)***

The mixture of ***9-trityl-9H-pyrrolo[2,3-b:4,5-c']dipyridine*** and ***2-(3-fluoropyridin-2-yl)-9-trityl-9H-pyrrolo[2,3-b:4,5-c']dipyridine*** (0.0586 g) was dissolved in dichloromethane (5 mL) and trifluoroacetic acid (1.8 mL) was added. The reaction mixture was stirred at room temperature for 6 hours. The reaction mixture was diluted with dichloromethane (50 mL) and water (20 mL). The pH of the aqueous phase was adjusted to pH~12 by the addition of a 1 M aqueous sodium hydroxide solution. The aqueous layer was separated, extracted with dichloromethane (25 mL), the combined organic layer dried over Na_2_SO_4_, filtered and the solvent removed under reduced pressure. The residue was purified by chromatography on silica (10 g HP-SIL) using a Biotage Isolera system employing a dichloromethane/methanol gradient (100/0 -> 95/5 -> 90/10) to afford more polar **12** as off-white solid (0.0067 g, 5.7 % for 3 steps). ^1^H-NMR (400 MHz, DMSO-d_6_) δ = 12.47 (br-s, 1H), 9.45 (s, 1H), 8.80 (d, *J*=8.1 Hz, 1H), 8.62 (d, *J*=4.5 Hz, 1H), 8.54 (d, *J*=5.8 Hz, 1H), 8.00 (d, *J*=8.1 Hz, 1H), 7.94-7.88 (m, 1H), 7.63-7.58 (m, 1H), 7.52 (d, *J*=5.6 Hz, 1H); MS (ESI): m/z = 264.84 [M+H]^+^.

**S14:** ^1^H-NMR spectra (DMSO-d_6_) of 2-(3-fluoropyridin-2-yl)-9*H*-pyrrolo[2,3-*b*:4,5-*c*']dipyridine (**12**)

**Synthesis of tritium labeling precursor compound 25**

***7-chloro-2-(3-chloro-2-fluoropyridin-4-yl)-9-trityl-9H-pyrrolo[2,3-b:4,5-c']dipyridine (25)***

***Step a:*** ***6'-bromo-6-chloro-2'-fluoro-[3,3'-bipyridin]-4-amine (22)***

A mixture of 1,4-dioxane (4 mL) and 2 M aqueous potassium phosphate solution (2 mL, 4 mmol) was degassed in a microwave vial for 3 minutes under sonication by passing a stream of argon through the mixture. Then commercially available 2-chloro-5-iodo-pyridine-4-amine **21** (0.509 g, 2 mmol), (6-bromo-2-fluoropyridin-3-yl)boronic acid **20** (0.438 g, 2 mmol) and [1,1′-bis(diphenylphosphino)-ferrocene]dichloropalladium(II) complex with dichloromethane (0.0815 g, 0.099 mmol) were added. The reaction mixture was heated at ~80°C in a sand-bath for 5 hours. The reaction mixture was diluted with ethyl acetate (100 mL), water (15 mL) and brine (15 mL). The organic phase was separated, dried over Na_2_SO_4_, filtered and the solvents were evaporated *in vacuo*. The black residue was purified by chromatography on silica (25 g, puriFlash, Interchim) using a Biotage Isolera system employing an ethyl acetate/n-heptane gradient (5/95 -> 10/90 -> 20/80 -> 30/70 -> 40/60 -> 60/40) to recover unreacted 2-chloro-5-iodo.pyridine-4-amine (0.343 g, 67 %) and to afford the more polar compound **22** as pale yellow solid (0.032 g, 5.3 %). MS (ESI): m/z = 302.2 (80 %), 304.2 (100%), 306.3 (25%) [M+H]^+^.

***Step b:***  ***2-bromo-7-chloro-9H-pyrrolo[2,3-b:4,5-c']dipyridine (23)***

Compound **22** (0.079 g, 0.262 mmol) was treated with a 1 M solution of lithium-bis(trimethylsilyl)amid in tetrahydrofurane (3 mL, 3 mmol) at 0 °C. The reaction mixture was heated at ~90 °C in a sand-bath for 2 hours. The cooled reaction mixture was added to water (50 mL), and the aqueous phase was extracted with ethyl acetate (2 x 50 mL). The organic phase was separated, dried over Na_2_SO_4_, filtered and the solvents were evaporated *in vacuo*. The dark residue was purified by chromatography on silica (25 g, HP-Ultra) using a Biotage Isolera system employing an ethyl acetate/n-heptane gradient (5/95 -> 10/90 -> 20/80 -> 30/70 -> 40/60 -> 60/40 -> 100/0) to afford compound **23** as a pale yellow solid (0.0484 g, 65 %). ^1^H-NMR (400 MHz, DMSO-d_6_) δ = 12.68 (br-s, 1H), 9.23 (s, 1H), 8.60 (d, 1H), 7.59-7.54 (m, 2H); MS (ESI): m/z = 282.2 (80 %), 284.1 (100%), 285.8 (25%), 286.2 (25%) [M+H]^+^.


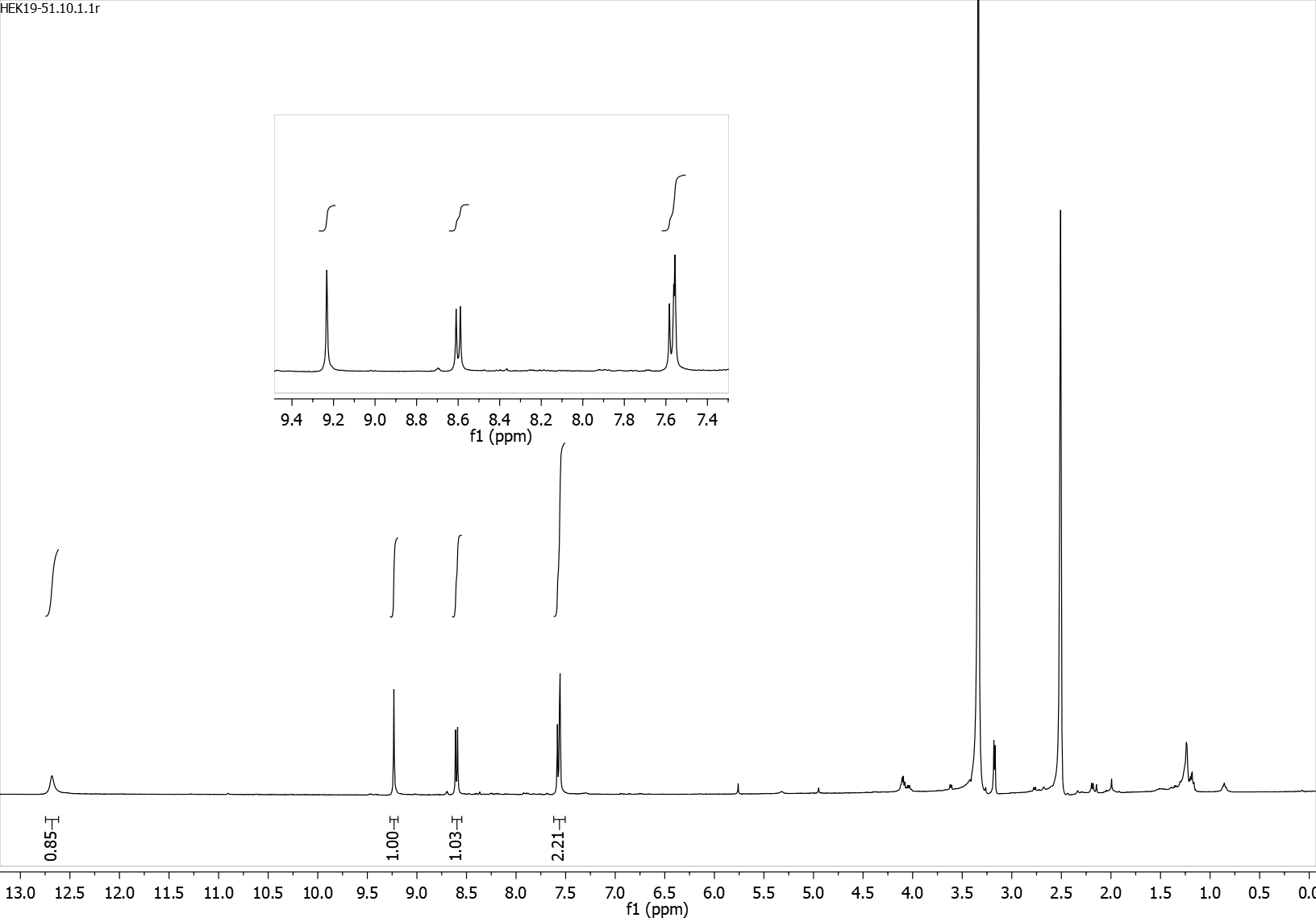


**S15:** ^1^H-NMR spectra (DMSO-d_6_) of 2-bromo-7-chloro-9*H*-pyrrolo[2,3-*b*:4,5-*c*']dipyridine (**23**)

***Step c: 2-bromo-7-chloro-9-trityl-9H-pyrrolo[2,3-b:4,5-c']dipyridine (24)***

To a suspension of compound **23** (0.043 g, 0.15 mmol) in dichloromethane (5 mL) was added triethylamine (0.092 mL, 0.64 mmol) and trityl-chloride (0.13 g, 0.463 mmol). After the addition of 4-(dimethylamino)-pyridine (0.0036 g, 0.03 mmol), the reaction mixture was stirred at room temperature for 16 hours. The reaction mixture was diluted with dichloromethane (80 mL) and water (20 mL). The organic phase was separated, dried over Na_2_SO_4_, filtered and the solvents were removed in *vacuo*. The residue was purified by chromatography on silica (12 g, puriFlash, Interchim) using a Biotage Isolera One purification system employing an ethyl acetate/n-heptane gradient (5/95 -> 10/90 -> 20/80 -> 30/70 -> 40/60) to afford compound **24** as a white solid (0.049 g, 61 %). ^1^H-NMR (400 MHz, CDCl_3_) δ = 8.96 (s, 1H), 8.10 (d, 1H), 7.48-7.40 (m, 6H), 7.36-7.25 (m, 10H), 6.28 (s, 1H); MS (ESI): m/z = 524.47/526.55 [M+H]^+^.


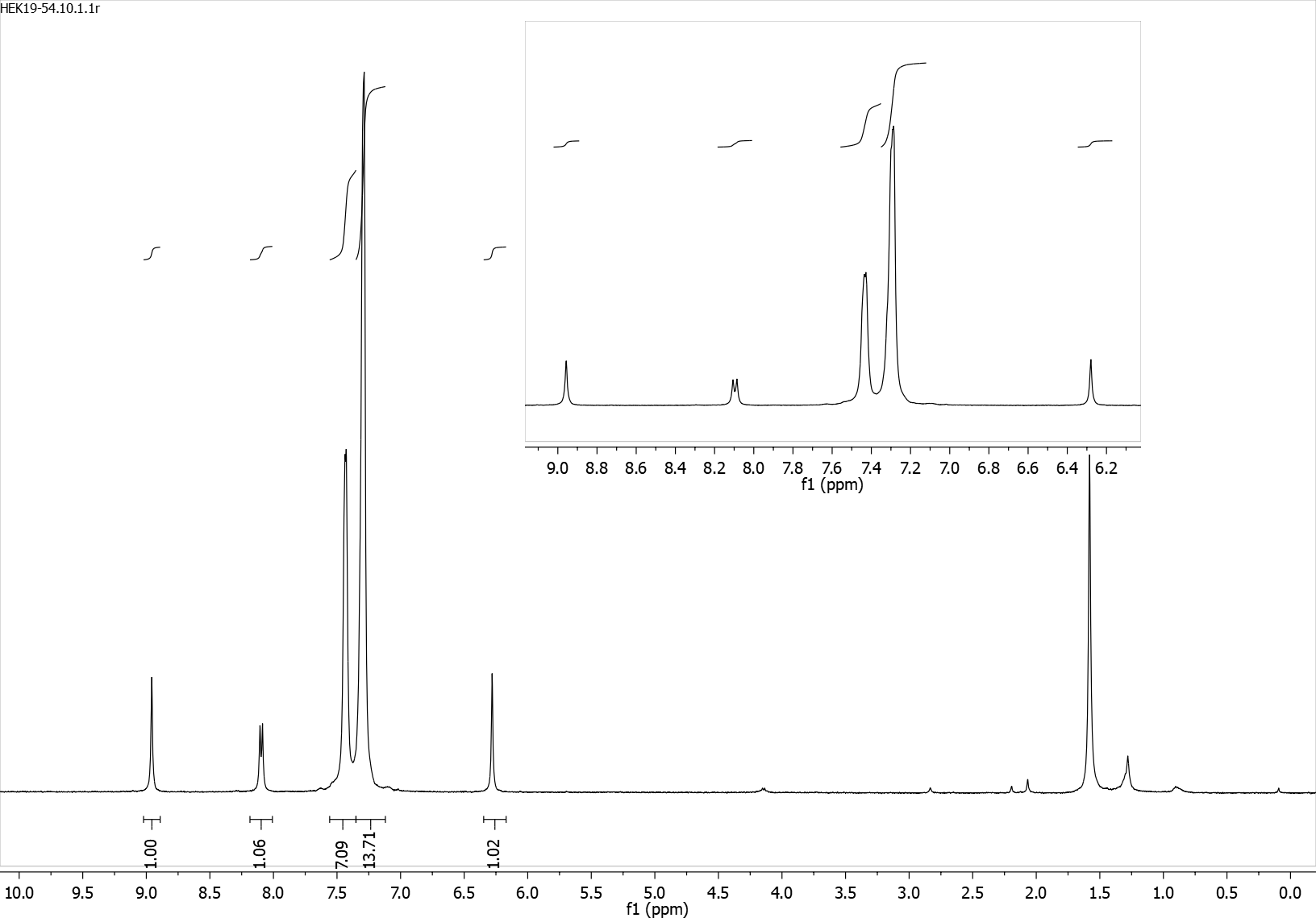


**S16:** ^1^H-NMR spectra (CDCl_3_) of 2-bromo-7-chloro-9-trityl-9*H*-pyrrolo[2,3-*b*:4,5-*c*']- dipyridine (**24**)

***Step d: 7-chloro-2-(3-chloro-2-fluoropyridin-4-yl)-9-trityl-9H-pyrrolo[2,3-b:4,5-c']- dipyridine (25)***

To a mixture of degassed 1,4-dioxane (1.9 mL) and water (0.45 mL) in a microwave vial was added [1,1′-bis(diphenylphosphino)ferrocene]dichloropalladium(II) complex with dichloromethane (0.0036 g, 0.0043 mmol), followed by compound **24** (0.0446 g, 0.085 mmol), (3-chloro-2-fluoropyridin-4-yl)boronic acid (0.0185 g, 0.106 mmol) and cesium carbonate (0.058 g, 0.156 mmol). The reaction mixture was then heated at ~120°C in a sand-bath for 6 hours. The reaction mixture was diluted with ethyl acetate (50 mL) and water (20 mL), the organic phase was separated, dried over Na_2_SO_4_, filtered and the solvents were evaporated *in vacuo*. The dark residue was purified by chromatography on silica (12 g, puriFlash, Interchim) using a Biotage Isolera system employing an ethyl acetate/n-heptane gradient (5/95 -> 10/90 -> 20/80 -> 30/70 -> 40/60 -> 60/40) to afford compound **25** as a white solid (0.0176 g, 36 %). ^1^H-NMR (400 MHz, CDCl_3_) δ = 9.04 (s, 1H), 8.41 (d, 1H), 7.92-7.86 (m, 2H), 7.50.7.45 (m, 6H), 7.30-7.26 (m, 9H), 6.73 (d, 1H), 6.29 (s, 1H); MS (ESI): m/z = 575.31/576.08/577.62 [M+H]^+^.


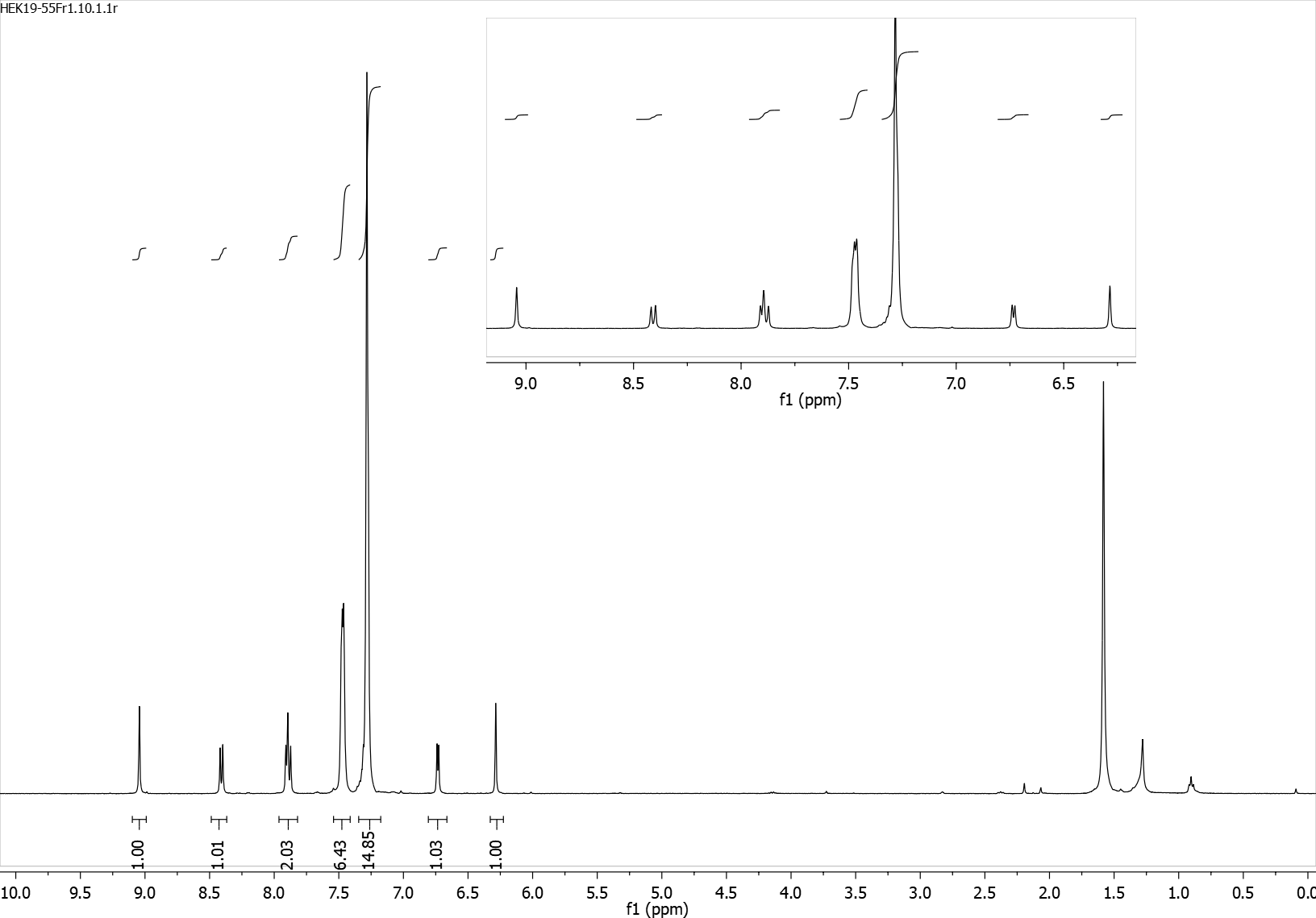


**S17:** ^1^H-NMR spectra (CDCl_3_) of 7-chloro-2-(3-chloro-2-fluoropyridin-4-yl)-9-trityl- 9*H*-pyrrolo[2,3-*b*:4,5-*c*']dipyridine (**25**)

**Experimental procedure and analytical characterization of [^3^H]PI-2620**

***2-(2-fluoro-3-tritiumpyridin-4-yl)-7-tritium-9H-pyrrolo[2,3-b:4,5-c']dipyridine ([3H]PI-2620)***

To the tritium reaction vessel was added 10% palladium on charcoal (0.003 g), followed by a solution of compound **25** (0.003 g, 0.0052 mmol) in *N*,*N*’-dimethylformamide (0.3 mL) and *N*,*N*-diisopropylethylamine (0.005 mL). The vessel was attached to the tritium line and pressurized to 0.5 atm with tritium gas at -200 °C. The solution was stirred for 8 hours at room temperature, cooled to -200 °C and excess tritium removed. The reaction flask was rinsed with methanol (4 x 1 mL) while passing each of the methanol washes through a celite pad. The combined filtrate was evaporated under reduced pressure to yield the tritium exchange product containing the trityl-protecting group (31 mCi). This material was purified by HPLC, the trityl-moiety cleaved with acetonitrile/trifluoro acetic acid, and again purified by HPLC. The mobile phase was removed under reduced pressure and the title compound redissolved in ethanol (yield: 12 mCi, purity >99 %). The specific activity was determined to be 46.56 Ci/mmol by MS (m/z = 265.40 (30 %), 267.43 (80 %), 269.45 (100 %), 271.43 (45 %).

HPLC: Eclipse XDB-C18, 5 μm, 4.6 x 150 mm; mobile phase A: 0.05% TFA/water; mobile phase B: acetonitrile; flow rate: 1.0 mL/min; gradient: t=0 (10% B), t=10 minutes (90% B), t=12 minutes (100% B).

**S18:** MS spectra of 2-(2-fluoro-3-tritiumpyridin-4-yl)-7-tritium-9*H*-pyrrolo-[2,3-*b*:4,5-*c*']dipyridine (**[^3^H]PI-2620**)

**S19:** HPLC chromatogram of 2-(2-fluoro-3-tritiumpyridin-4-yl)-7-tritium-9*H*-pyrrolo-[2,3-*b*:4,5-*c*']dipyridine (**[^3^H]PI-2620**)

**Experimental procedures, analytical characterization and ^1^H-NMR spectra of corresponding nitro-precursor compounds to prepare [^18^F]-labeled compounds 3-12**

**Nitro-precursor compound 26 to prepare [^18^F]7**

***2-(2-nitropyridin-4-yl)-9-trityl-9*H*-pyrrolo[2,3-*b*:4,5-*c*']dipyridine (26)***

To a mixture of degassed 1,4-dioxane (4.3 mL) and water (1 mL) in a microwave vial was added [1,1′-bis(diphenylphosphino)ferrocene]dichloropalladium(II), complex with dichloromethane (0.0084 g, 0.01 mmol), followed by compound **17** (0.1 g, 0.2 mmol), commercially available 2-nitro-4-(4,4,5,5-tetramethyl-1,3,2-dioxaborolan-2-yl)pyridine (0.061 g, 0.245 mmol) and cesium carbonate (0.133 g, 0.41 mmol). The reaction mixture was then heated at ~115°C in a sand-bath for 6 hours. The reaction mixture was diluted with ethyl acetate (60 mL) and water (20 mL), the organic phase was separated, dried over Na_2_SO_4_, filtered and the solvents were evaporated *in vacuo*. The dark residue was purified by chromatography on silica (25 g pufiFlash-column, Interchim) using a Biotage Isolera system employing an ethyl acetate/n-heptane gradient (5/95 -> 100/0 -> 100/0) to afford **26** as a pale-yellow solid (0.082 g, 75 %). ^1^H-NMR (400 MHz, CDCl_3_) δ = 9.32 (s, 1H); 8.56 (d, *J*=5.0 Hz, 1H), 8.48 (d, *J*=8.0 Hz, 1H), 8.33 (s, 1H); 8.30 (d, *J*=6.1 Hz, 1H), 7.85 (d, *J*=8.0 Hz, 1H), 7.69 (d, *J*=5.0 Hz, 1H), 7.58-7.54 (m, 5H), 7.32-7.25 (m, 10H), 6.48 (d, *J*=6.1 Hz, 1H); MS (ESI): m/z = 534.28 [M+H]^+^.

**S20:** ^1^H-NMR spectra (CDCl_3_) of 2-(2-nitropyridin-4-yl)-9-trityl-9*H*-pyrrolo[2,3-*b*:4,5-*c*']dipyridine (**26**)

**Nitro-precursor compound 27 to prepare [^18^F]4**

***2-(2-nitropyridin-3-yl)-9-trityl-9*H*-pyrrolo[2,3-*b*:4,5-*c*']dipyridine (27)***

To a mixture of degassed 1,4-dioxane (8 mL) in a microwave vial was added [1,1′-bis(diphenylphosphino)ferrocene]dichloropalladium(II), complex with dichloromethane (0.034 g, 0.04 mmol), followed by compound **17** (0.2 g, 0.4 mmol), bis(pinacolato)diborane (0.112 g, 0.44 mmol) and potassium acetate (0.118 g, 1.2 mmol). The reaction mixture was then heated at ~95°C in a sand-bath for 18 hours. The reaction mixture was diluted with ethyl acetate (100 mL) and water (30 mL), the organic phase separated, dried over Na_2_SO_4_, filtered and the solvents evaporated *in vacuo* to afford the crude boronic ester derivative. The crude boronic ester derivative was dissolved in a mixture of degassed 1,4-dioxane (8.6 mL) and water (2 mL) in a microwave vial. Then [1,1′-bis(diphenylphosphino)ferrocene]dichloro-palladium(II), complex with dichloromethane (0.034 g, 0.04 mmol), 3-bromo-2-nitropyridine (0.1 g, 0.49 mmol) and cesium carbonate (0.266 g, 0.82 mmol) were added and the reaction mixture was heated at ~115°C in a sand-bath for 6 hours. The reaction mixtures of 4 runs were combined and diluted with ethyl acetate (600 mL) and water (200 mL), the organic phase separated, dried over Na_2_SO_4_, filtered and the solvents evaporated *in vacuo*. The dark residue was split in two portions and each portion was purified by chromatography on silica (40 g pufiFlash-column, Interchim) using a Biotage Isolera system employing an ethyl acetate/n-heptane gradient (5/95 -> 100/0 -> 100/0) to afford a mixture of the more polar compound **27** and a less polar byproduct. The mixture (0.260 g) was further purified by preparative TLC with a loading of ~0.03 g of mixture per 1000 μM Analtech Uniplate (20 x 20 cm) using ethyl acetate/n-heptane (70/30) as mobile phase to afford the more polar compound **27** as pale yellow solid (0.1 g, 11.8 %). ^1^H-NMR (400 MHz, CDCl_3_) δ = 9.28 (s, 1H), 8.47 (d, *J*=4.5 Hz, 1H); 8.38 (d, *J*=8.0 Hz, 1H), 8.26 (d, *J*=6.1 Hz, 1H), 7.55-7.53 (m, 1H). 7.52-7.46 (m, 6H), 7.32-7.20 (m, 11 H), 6.46 (d, *J*=6.1 Hz, 1H); MS (ESI): m/z = 534.17 [M+H]^+^.

**S21:** ^1^H-NMR spectra (CDCl_3_) of 2-(2-nitropyridin-3-yl)-9-trityl-9*H*-pyrrolo[2,3-*b*:4,5-*c*']dipyridine (**27**)

**Nitro-precursor compound 28 to prepare [^18^F]3**

To a mixture of degassed 1,4-dioxane (3 mL) and water (0.7 mL) in a microwave vial was added [1,1′-bis(diphenylphosphino)ferrocene]dichloropalladium(II), complex with dichloro-methane (0.0058 g, 0.007 mmol), followed by compound **18** (0.05 g, 0.143 mmol), 2-nitro-5-(4,4,5,5-tetramethyl-1,3,2-dioxaborolan-2-yl)pyridine (0.0428 g, 0.17 mmol) and cesium carbonate (0.092 g, 0.286 mmol). The reaction mixture was then heated at ~100°C in a sand-bath for 4 hours. The reaction mixture was diluted with ethyl acetate (80 mL) and water (35 mL), the organic phase separated, dried over Na_2_SO_4_, filtered and the solvents evaporated *in vacuo*. The dark residue was purified by chromatography on silica (12 g, puriFlash, Interchim) using a Biotage Isolera system employing a dichloromethane/methanol gradient (100/0 -> 98/2 -> 95/5 -> 90/10 -> 80/20) to afford **28** as a pale yellow solid (0.0173 g, 31%). ^1^H-NMR (400 MHz, CDCl_3_/CD_3_OD) δ = 9.45 (dd, *J*=2.3 Hz, *J*=0.8 Hz, 1H), 9.32 (d, *J*=0.8 Hz, 1H), 8.93 (dd, *J*=8.5 Hz, *J*=2.3 Hz, 1H), 8.68-8.64 (m, 2H), 8.46 (d, *J*=8.5 Hz, 1H), 8.35 (dd, *J*=5.9 Hz, *J*=1.0 Hz, 1H), 8.14 (d, *J*=8.1 Hz, 1H), 1.82 (s, 9H); MS (ESI): m/z = 392.13 [M+H]^+^.

**S22:** ^1^H-NMR spectra (CDCl_3_/CD_3_OD) of *tert*-butyl 2-(6-nitropyridin-3-yl)-9*H*-pyrrolo[2,3-*b*:4,5-*c*']dipyridine-9-carboxylate (**28**)

**Nitro-precursor compound 29 to prepare [^18^F]5**

To a mixture of degassed 1,4-dioxane (8 mL) in a microwave vial was added [1,1′-bis(diphenylphosphino)ferrocene]dichloropalladium(II), complex with dichloromethane (0.034 g, 0.04 mmol), followed by compound **17** (0.2 g, 0.4 mmol), bis(pinacolato)diborane (0.112 g, 0.44 mmol) and potassium acetate (0.118 g, 1.2 mmol). The reaction mixture was then heated at ~95°C in a sand-bath for 18 hours. The reaction mixture was diluted with ethyl acetate (100 mL) and water (30 mL), the organic phase separated, dried over Na_2_SO_4_, filtered and the solvents evaporated *in vacuo* to afford the crude boronic ester derivative. The crude boronic ester derivative was dissolved in a mixture of degassed 1,4-dioxane (8.6 mL) and water (2 mL) in a microwave vial. Then [1,1′-bis(diphenylphosphino)ferrocene]dichloro-palladium(II), complex with dichloromethane (0.034 g, 0.04 mmol), 3-bromo-5-nitropyridine (0.05 g, 0.245 mmol) and cesium carbonate (0.133 g, 0.41 mmol) were added and the reaction mixture was heated at ~115°C in a sand-bath for 6 hours. The reaction mixture was diluted with ethyl acetate (80 mL) and water (30 mL), the organic phase separated, dried over Na_2_SO_4_, filtered and the solvents evaporated *in vacuo*. The dark residue was purified by chromatography on silica (25 g puriFlash, Interchim) using a Biotage Isolera system employing an ethyl acetate/n-heptane gradient (5/95 -> 100/0 -> 100/0) to afford **29** as pale yellow solid (0.0144 g, 13 %). ^1^H-NMR (400 MHz, CDCl_3_) δ = 9.36 (br-s, 1H), 9.30 (br-s, 1H), 9.02 (br-s, 1H); 8.52-8.48 (m, 2H), 8.29 (d, *J*=5.8 Hz, 1H), 7.80 (d, *J*=8.0 Hz, 1H), 7.60-7.55 (m, 5H), 7.33-7.25 (m, 10H), 6.46 (d, *J*=6.1 Hz, 1H); MS (ESI): m/z = 533.67 [M+H]^+^.

**S23:** ^1^H-NMR spectra (CDCl_3_) of 2-(5-nitropyridin-3-yl)-9-trityl-9*H*-pyrrolo[2,3-*b*:4,5-*c*']dipyridine (**29**)

**Nitro-precursor compound 30 to prepare [^18^F]8**

To a mixture of degassed 1,4-dioxane (8 mL) in a microwave vial was added [1,1′-bis(diphenylphosphino)ferrocene]dichloropalladium(II), complex with dichloromethane (0.034 g, 0.04 mmol), followed by compound **17** (0.2 g, 0.4 mmol), bis(pinacolato)diborane (0.112 g, 0.44 mmol) and potassium acetate (0.118 g, 1.2 mmol). The reaction mixture was then heated at ~95°C in a sand-bath for 18 hours. The reaction mixture was diluted with ethyl acetate (100 mL) and water (30 mL), the organic phase separated, dried over Na_2_SO_4_, filtered and the solvents evaporated *in vacuo* to afford the crude boronic ester derivative. The crude boronic ester derivative was dissolved in a mixture of degassed 1,4-dioxane (8.6 mL) and water (2 mL) in a microwave vial. Then [1,1′-bis(diphenylphosphino)ferrocene]dichloro-palladium(II), complex with dichloromethane (0.034 g, 0.04 mmol), 4-chloro-3-nitro-pyridine (0.078 g, 0.49 mmol) and cesium carbonate (0.266 g, 0.82 mmol) were added and the reaction mixture was heated at ~115°C in a sand-bath for 6 hours. The reaction mixture was diluted with ethyl acetate (80 mL) and water (30 mL), the organic phase was separated, dried over Na_2_SO_4_, filtered and the solvents were evaporated *in vacuo*. The dark residue was purified by chromatography on silica (25 g puriFlash, Interchim) using a Biotage Isolera system employing an ethyl acetate/n-heptane gradient (5/95 -> 100/0 -> 100/0) to afford **30** as a pale yellow solid (0.033 g, 15 %). ^1^H-NMR (400 MHz, CDCl_3_) δ = 9.30 (s, 1H), 9.02 (s, 1H), 8.68 (d, *J*=5.1 Hz, 1H), 8.42 (d, *J*=8.0 Hz, 1H), 8.26 (d, *J*=6.1 Hz, 1H), 7.49-7.45 (m, 5H), 7.31 (d, *J*=8.1 Hz, 1H), 7.27-7.22 (m, 10H); 7.08 (d, *J*=5.0 Hz, 1H), 6.44 (d, *J*=6.1 Hz, 1H); MS (ESI): m/z = 533.59 [M+H]^+^.

**S24:** ^1^H-NMR spectra (CDCl_3_) of 2-(3-nitropyridin-4-yl)-9-trityl-9*H*-pyrrolo[2,3-*b*:4,5-*c*']dipyridine (**30**)

**Nitro-precursor compound 31 to prepare [^18^F]6**

In a 20 ml microwave tube was dissolved compound **17** (0.2 g, 0.408 mmol) and 4-nitro-3-(4,4,5,5-tetramethyl-1,3,2-dioxaborolan-2-yl)pyridine (0.204 g, 0.816 mmol) in *N*,*N*’-dimethylacetamide (5.10 mL). Sodium carbonate (0.816 ml, 1.631 mmol) was added and the resulting stirring solution was degassed for 5 minutes. Then [1,1′-bis(diphenylphosphino)ferrocene]dichloropalladium(II) complex with dichloromethane was added (0.017 g, 0.02 mmol) and the reaction mixture was heated to 110°C for 22 hours. TLC monitoring showed completion of the reaction. The reaction mixture was diluted with dichloromethane, insolubles were filtered out through Celite, and the filtrate was washed with water three times to remove residual amounts of *N*,*N*’-dimethylacetamide. The organic layer was dried MgSO_4_, filtered and concentrated. The residue was purified via Biotage Isolera One employing an ethyl acetate/n-heptane gradient (5/95 -> 100/0 -> 100/0) to afford **31** as pale yellow solid (0.056 g, 28 %). ^1^H-NMR (400 MHz, DMSO-d_6_) δ = 9.45 (s, 1H), 8.81 (d, *J*=8.1 Hz, 1H), 8.69 (d, *J*=2.2 Hz, 1H), 8.32-8.23 (m, 3H), 8.20 (d, *J*=8.1 Hz, 1H), 7.62-7.58 (m, 6H), 7.36-7.22 (m, 9H), 6.52 (d, *J*=6.1 Hz, 1H); MS (ESI): m/z = 533.87 [M+H]^+^.

**S25:** ^1^H-NMR spectra (DMSO-d_6_) of 2-(4-nitropyridin-3-yl)-9-trityl-9*H*-pyrrolo[2,3-*b*:4,5-*c*']dipyridine (**31**)

**Nitro-precursor compound 32 to prepare [^18^F]9**

To a mixture of degassed 1,4-dioxane (8 mL) in a microwave vial was added [1,1′-bis(diphenylphosphino)ferrocene]dichloropalladium(II), complex with dichloromethane (0.034 g, 0.04 mmol), followed by compound **17** (0.2 g, 0.4 mmol), bis(pinacolato)diborane (0.112 g, 0.44 mmol) and potassium acetate (0.118 g, 1.2 mmol). The reaction mixture was then heated at ~95°C in a sand-bath for 18 hours. The reaction mixture was diluted with ethyl acetate (100 mL) and water (30 mL), the organic phase separated, dried over Na_2_SO_4_, filtered and the solvents evaporated *in vacuo* to afford the crude boronic ester derivative. In a 20 ml microwave tube was dissolved the boronic ester derivative, 2-bromo-6-nitropyridine (0.05 g, 0.245 mmol) in *N*,*N*’-dimethylacetamide (5.10 mL). Sodium carbonate (0.408 ml, 0.816 mmol) was added and the resulting stirring solution was degassed for 5 minutes. Then [1,1′-bis(diphenylphosphino)ferrocene]dichloropalladium(II) complex with dichloromethane was added (0.017 g, 0.02 mmol) and the reaction mixture was heated to 110°C for 22 hours. TLC monitoring showed completion of the reaction. The reaction mixture was diluted with dichloromethane, insolubles were filtered out through Celite, and the filtrate was washed with water three times to remove residual amounts of *N*,*N*’-dimethylacetamide. The organic layer was dried MgSO_4_, filtered and concentrated. The residue was purified via Biotage Isolera One employing an ethyl acetate/n-heptane gradient (5/95 -> 100/0 -> 100/0) to afford a mixture of **32** and dehalogenated **17** (ratio: 60/40) as pale yellow solid (0.0174 g, 16 %). ^1^H-NMR (400 MHz, DMSO-d_6_) δ = 9.43 (s, 1H), 9.38 (s, 1H), 8.81 (d, *J*=8.1 Hz, 1H), 8.60 (dd, *J*=7.8 Hz, *J*=1.6 Hz, 1H), 8.33 (d, *J*=7.8 Hz, 1H), 8.28-8.24 (m, 2H), 8.19-8.17 (m, 1H), 8.10 (t, *J*=7.0 Hz, 1H), 7.63-759 (m, 7H), 7.49-745 (m, 4H), 7.42 (d, *J*=7.8 Hz, 1H), 7.34-7.21 (m, 18H), 6.58 (d, *J*=6.0 Hz, 1H), 6.19 (d, *J*=6.1 Hz, 1H); MS (ESI): m/z = 533.62 [M+H]^+^.

**S26:** ^1^H-NMR spectra (DMSO-d_6_) of 2-(6-nitropyridin-2-yl)-9-trityl-9*H*-pyrrolo-[2,3-*b*:4,5-*c*']dipyridine (**32**)

**Nitro-precursor compound 33 to prepare [^18^F]10**

To a mixture of degassed 1,4-dioxane (8 mL) in a microwave vial was added [1,1′-bis(diphenylphosphino)ferrocene]dichloropalladium(II), complex with dichloromethane (0.034 g, 0.04 mmol), followed by compound **17** (0.2 g, 0.4 mmol), bis(pinacolato)diborane (0.112 g, 0.44 mmol) and potassium acetate (0.118 g, 1.2 mmol). The reaction mixture was then heated at ~95°C in a sand-bath for 18 hours. The reaction mixture was diluted with ethyl acetate (100 mL) and water (30 mL), the organic phase separated, dried over Na_2_SO_4_, filtered and the solvents evaporated *in vacuo* to afford the crude boronic ester derivative. The crude boronic ester derivative was dissolved in a mixture of degassed 1,4-dioxane (8.6 mL) and water (2 mL) in a microwave vial. Then [1,1′-bis(diphenylphosphino)ferrocene]dichloro-palladium(II), complex with dichloromethane (0.034 g, 0.04 mmol), 2-bromo-5-nitropyridine (0.1 g, 0.49 mmol) and cesium carbonate (0.266 g, 0.82 mmol) were added and the reaction mixture was heated at ~115°C in a sand-bath for 6 hours. The reaction mixture was diluted with ethyl acetate (100 mL) and water (30 mL), the organic phase separated, dried over Na_2_SO_4_, filtered and the solvents evaporated *in vacuo*. The dark residue was purified by chromatography on silica (25 g puriFlash, Interchim) using a Biotage Isolera system employing an ethyl acetate/n-heptane gradient (5/95 -> 100/0 -> 100/0) to afford a mixture of two compounds (0.0788 g). The mixture of two compounds was dissolved in dichloromethane (10 mL) and trifluoroacetic acid (2.4 mL) was added. The reaction mixture was stirred at room temperature for 6 hours and then methanol was added (10 mL). The solvents were evaporated *in vacuo* and the residue suspended in methanol (10 mL). The solvents were again evaporated *in vacuo* and the residue suspended in dichloromethane (4 mL). After the addition of triethylamine (2 mL, 14.4 mmol), di-*tert*-butyl dicarbonate (0.2 g, 0.86 mmol), and 4-(dimethylamino)-pyridine (0.0036 g, 0.028 mmol), the reaction mixture was stirred at room temperature for 18 hours. The reaction mixture was diluted with ethyl acetate (100 mL) and water (40 mL). The organic phase was separated, dried over Na_2_SO_4_, filtered and the solvents removed in *vacuo*. The residue was purified on silica (25 g puriFlash, Interchim) using a Biotage Isolera One purification system employing an ethyl acetate/n-heptane gradient (5/95 -> 100/0 -> 100/0) to afford **33** as pale yellow solid (0.0149 g, 25.7 %). ^1^H NMR (400 MHz, CDCl_3_) δ = 9.55 (d, *J*=2.6 Hz, 1H), 9.36 (s, 1H), 8.88 (d, *J*=8.1 Hz, 1H), 8.77 (d, *J*=8.8 Hz, 1H), 8.72 (d, *J*=5.9 Hz, 1H), 8.65 (dd, *J*=8.8 Hz, *J*=2.6 Hz, 1H), 8.56 (d, *J*=8.1 Hz, 1H), 8.30 (d, *J*=5.9 Hz, 1H), 1.87 (s, 9H); MS (ESI): m/z = 391.93 [M+H]^+^.

**S27:** ^1^H-NMR spectra (CDCl_3_) of *tert*-butyl 2-(5-nitropyridin-2-yl)-9*H*-pyrrolo-[2,3-*b*:4,5-*c*']dipyridine-9-carboxylate (**33**)

**Nitro-precursor compound 34 to prepare [^18^F]11**

To a mixture of degassed 1,4-dioxane (8 mL) in a microwave vial was added [1,1′-bis(diphenylphosphino)ferrocene]dichloropalladium(II), complex with dichloromethane (0.034 g, 0.04 mmol), followed by compound **17** (0.2 g, 0.4 mmol), bis(pinacolato)diborane (0.112 g, 0.44 mmol) and potassium acetate (0.118 g, 1.2 mmol). The reaction mixture was then heated at ~95°C in a sand-bath for 18 hours. The reaction mixture was diluted with ethyl acetate (100 mL) and water (30 mL), the organic phase separated, dried over Na_2_SO_4_, filtered and the solvents evaporated *in vacuo* to afford the crude boronic ester derivative. The crude boronic ester derivative was dissolved in a mixture of degassed 1,4-dioxane (8.6 mL) and water (2 mL) in a microwave vial. Then [1,1′-bis(diphenylphosphino)ferrocene]dichloro-palladium(II), complex with dichloromethane (0.034 g, 0.04 mmol), 2-bromo-4-nitropyridine (0.1 g, 0.49 mmol) and cesium carbonate (0.266 g, 0.82 mmol) were added and the reaction mixture was heated at ~115°C in a sand-bath for 6 hours. The reaction mixture was diluted with ethyl acetate (100 mL) and water (30 mL), the organic phase separated, dried over Na_2_SO_4_, filtered and the solvents evaporated *in vacuo*. The dark residue was purified by chromatography on silica (25 g puriFlash, Interchim) using a Biotage Isolera system employing an ethyl acetate/n-heptane gradient (5/95 -> 100/0 -> 100/0) to afford a mixture of compounds (0.076 g). The mixture of compounds was dissolved in dichloromethane (10 mL) and trifluoroacetic acid (2.4 mL) was added. The reaction mixture was stirred at room temperature for 6 hours and then methanol was added (10 mL). The solvents were evaporated *in vacuo* and the residue suspended in methanol (10 mL). The solvents were again evaporated *in vacuo* and the residue suspended in dichloromethane (4 mL). After the addition of triethylamine (2 mL, 14.4 mmol), di-*tert*-butyl dicarbonate (0.2 g, 0.86 mmol), and 4-(dimethylamino)-pyridine (0.0036 g, 0.028 mmol), the reaction mixture was stirred at room temperature for 18 hours. The reaction mixture was diluted with ethyl acetate (100 mL) and water (40 mL). The organic phase was separated, dried over Na_2_SO_4_, filtered and the solvents removed in *vacuo*. The residue was purified on silica (25 g puriFlash, Interchim) using a Biotage Isolera One purification system employing an ethyl acetate/n-heptane gradient (5/95 -> 100/0 -> 100/0) to afford **34** and dehalogenated **18** as ~1:1-mixture (0.0231 g, pale yellow solid). ^1^H NMR (400 MHz, CDCl_3_) δ = 9.38 (d, *J*=2.2 Hz, 1H), 9.35 (d, *J*=1.0 Hz, 1H), 9,31 (s, 2H), 9.02 (d, *J*=5.3 Hz, 1H), 8.76-8.70 (m, 5H), 8.68 (d, 1H), 8.55 (d, *J*=8.1 Hz, 1H), 8.43-8.37 (m, 3H), 8.12 (dd, *J*=5.8 Hz, *J*=1.0 Hz, 1H), 8.07 (dd, *J*=5.3 Hz, *J*=2.2 Hz, 1H), 7.43 (d, *J*=4.9 Hz, 1H), 7.41 (d, *J*=4.9 Hz, 1H), 1.82 (s, 18H); MS (ESI): m/z = 291.94 [MH-Boc]^+^ of **34**, 170.04 [MH^+^-Boc]^+^ of dehalogenated **18**.

**S28**: ^1^H-NMR spectra (CDCl_3_) of *tert*-butyl 2-(4-nitropyridin-2-yl)-9*H*-pyrrolo-[2,3-*b*:4,5-*c*']dipyridine-9-carboxylate (**34**)

**Nitro-precursor compound 35 to prepare [^18^F]12**

To a mixture of degassed 1,4-dioxane (8 mL) in a microwave vial was added [1,1′-bis(diphenylphosphino)ferrocene]dichloropalladium(II), complex with dichloromethane (0.034 g, 0.04 mmol), followed by compound **17** (0.2 g, 0.4 mmol), bis(pinacolato)diborane (0.112 g, 0.44 mmol) and potassium acetate (0.118 g, 1.2 mmol). The reaction mixture was then heated at ~95°C in a sand-bath for 18 hours. The reaction mixture was diluted with ethyl acetate (100 mL) and water (30 mL), the organic phase separated, dried over Na_2_SO_4_, filtered and the solvents evaporated *in vacuo* to afford the crude boronic ester derivative. The crude boronic ester derivative was dissolved in a mixture of degassed 1,4-dioxane (8.6 mL) and water (2 mL) in a microwave vial. Then [1,1′-bis(diphenylphosphino)ferrocene]dichloro-palladium(II), complex with dichloromethane (0.034 g, 0.04 mmol), 2-bromo-3-nitropyridine (0.1 g, 0.49 mmol) and cesium carbonate (0.266 g, 0.82 mmol) were added and the reaction mixture was heated at ~115°C in a sand-bath for 6 hours. The reaction mixture was diluted with ethyl acetate (100 mL) and water (30 mL), the organic phase separated, dried over Na_2_SO_4_, filtered and the solvents evaporated *in vacuo*. The dark residue was purified by chromatography on silica (25 g puriFlash, Interchim) using a Biotage Isolera system employing an ethyl acetate/n-heptane gradient (5/95 -> 100/0 -> 100/0) to afford a mixture of two compounds (0.0538 g). The mixture of two compounds was dissolved in dichloromethane (4 mL) and trifluoroacetic acid (2.5 mL) was added. The reaction mixture was stirred at room temperature for 16 hours and then methanol was added (10 mL). The solvents were evaporated *in vacuo* and the residue suspended in methanol (10 mL). The solvents were again evaporated *in vacuo* and the residue suspended in dichloromethane (4 mL). After the addition of triethylamine (2 mL, 14.4 mmol), di-*tert*-butyl dicarbonate (0.2 g, 0.86 mmol), and 4-(dimethylamino)-pyridine (0.0036 g, 0.028 mmol), the reaction mixture was stirred at room temperature for 18 hours. The reaction mixture was diluted with ethyl acetate (100 mL) and water (40 mL). The organic phase was separated, dried over Na_2_SO_4_, filtered and the solvents removed in *vacuo*. The residue was purified on silica (25 g puriFlash, Interchim) using a Biotage Isolera One purification system employing an ethyl acetate/n-heptane gradient (5/95 -> 100/0 -> 100/0) to afford **35** as pale yellow solid (0.0194 g, 12.1 % for 3 steps). ^1^H NMR (400 MHz, CDCl_3_) δ = 9.35 (d, 1H), 8.90 (d, *J*=4.8 Hz, 1H), 8.73 (d, *J*=5.8 Hz, 1H), 8.58 (d, *J*=8.1 Hz, 1H), 8.24-8.17 (m, 3H), 7.57-7.53 (m, 1H), 1.73 (s, 9H); MS (ESI): m/z = 391.92 [MH^+^], 291.90 [MH^+^-Boc].

**S29:** ^1^H-NMR spectra (CDCl_3_) of *tert*-butyl 2-(3-nitropyridin-2-yl)-9*H*-pyrrolo-[2,3-*b*:4,5-*c*']dipyridine-9-carboxylate (**35**)
